# Accurate and fast graph-based pangenome annotation and clustering with ggCaller

**DOI:** 10.1101/gr.277733.123

**Published:** 2023-09

**Authors:** Samuel T. Horsfield, Gerry Tonkin-Hill, Nicholas J. Croucher, John A. Lees

**Affiliations:** 1 MRC Centre for Global Infectious Disease Analysis, Department of Infectious Disease Epidemiology, Imperial College London, London W12 0BZ, United Kingdom;; 2 European Molecular Biology Laboratory, European Bioinformatics Institute, Wellcome Genome Campus, Hinxton CB10 1SD, United Kingdom;; 3 Department of Biostatistics, University of Oslo, Blindern, 0372 Oslo, Norway

## Abstract

Bacterial genomes differ in both gene content and sequence mutations, which underlie extensive phenotypic diversity, including variation in susceptibility to antimicrobials or vaccine-induced immunity. To identify and quantify important variants, all genes within a population must be predicted, functionally annotated, and clustered, representing the “pangenome.” Despite the volume of genome data available, gene prediction and annotation are currently conducted in isolation on individual genomes, which is computationally inefficient and frequently inconsistent across genomes. Here, we introduce the open-source software graph-gene-caller (ggCaller). ggCaller combines gene prediction, functional annotation, and clustering into a single workflow using population-wide de Bruijn graphs, removing redundancy in gene annotation and resulting in more accurate gene predictions and orthologue clustering. We applied ggCaller to simulated and real-world bacterial data sets containing hundreds or thousands of genomes, comparing it to current state-of-the-art tools. ggCaller has considerable speed-ups with equivalent or greater accuracy, particularly with data sets containing complex sources of error, such as assembly contamination or fragmentation. ggCaller is also an important extension to bacterial genome-wide association studies, enabling querying of annotated graphs for functional analyses. We highlight this application by functionally annotating DNA sequences with significant associations to tetracycline and macrolide resistance in *Streptococcus pneumoniae*, identifying key resistance determinants that were missed when using only a single reference genome. ggCaller is a novel bacterial genome analysis tool with applications in bacterial evolution and epidemiology.

Accurate representation of a population's genomic diversity, known as a pangenome, is critical in epidemiological and evolutionary studies of bacterial species. Identification of core genes, found in all individuals, is used for classification and epidemiological analyses. Such methods include phylogenetic analysis (Wolf et al. 2002; Croucher et al. 2013b; Zakham et al. 2021) and transmission chain inference during outbreaks (Ypma et al. 2013; Croucher and Didelot 2015). Genes present in only a subset of isolates, known as accessory genes, are often correlated with particular strains (Lees et al. 2019). These genes have also been associated with the wide phenotypic diversity found in many bacterial species, including antimicrobial resistance (AMR) (Jaillard et al. 2017; McNally et al. 2019), virulence (Alikhan et al. 2018; Hennart et al. 2020), host range (Weinert et al. 2015; Dearlove et al. 2016), and vaccine escape (Lo et al. 2019). Accessory genes are the focus of many evolutionary models of bacterial population structure and dynamics, such as understanding how multistrain populations emerge and are maintained (Baumdicker et al. 2012; Iranzo et al. 2019; Harrow et al. 2021) and predicting how they respond to perturbations such as vaccines (Corander et al. 2017; Azarian et al. 2020).

Pangenome studies rely on gene prediction in each isolate genome assembly followed by similarity-based clustering, generating clusters of orthologous genes (COGs). These steps are currently run as separate bioinformatic processes, split into gene prediction tools, or gene-callers, and pangenome analysis tools. Gene-callers, such as Glimmer (Delcher et al. 2007), Prodigal (Hyatt et al. 2010), GeneMarkS-2 (Lomsadze et al. 2018), and Balrog (Sommer and Salzberg 2021), predict the locations of coding sequences (CDSs) in individual genomes using models of gene sequence and gene overlap penalization. There has been little recent innovation in gene prediction algorithms; a comprehensive benchmarking study of existing tools included only one tool released in the past 10 years, and highlighted that no tool was universally applicable across bacteria (Dimonaco et al. 2022). Contrastingly, gene annotation, whereby gene prediction tools are integrated with annotation databases to assign functional labels to predicted genes, has seen increased attention. Popular examples include PGAP (Tatusova et al. 2016), Prokka (Seemann 2014), DFAST (Tanizawa et al. 2018), and Bakta (Schwengers et al. 2021). As gene prediction and annotation tools are designed for analyzing single genomes only, a key issue when applying these tools in pangenome studies is the consistency in prediction and annotations across orthologs. For example, if predicted start or stop positions vary between orthologs (termed a “prediction error”), underclustering can occur, whereby truly homologous genes do not share enough sequence to be placed in the same cluster (Zhou et al. 2020; Tonkin-Hill et al. 2023). Orthologs may also be given inconsistent functional annotations (termed an “annotation error”), leading to ambiguity during functional inference of gene families (Tonkin-Hill et al. 2020). Moreover, functional annotations are applied to genes individually, generating huge computational redundancy, as orthologs are annotated in each genome rather than once within the population. This leads to increased runtime, ultimately limiting the size and therefore comprehensiveness of the annotation database that can be used (Schwengers et al. 2021). Finally, poor assembly quality, such as contamination and fragmentation, can impact gene prediction accuracy by introducing false-positive predictions, such as contaminant genes or partial gene sequences (Tonkin-Hill et al. 2020; Tonkin-Hill et al. 2023). Gene prediction and annotation, specifically for pangenome studies, require innovations to ensure orthologs are identified and annotated consistently across a population.

Pangenome analysis tools cluster the predicted gene sequences from all input genomes, representing the pangenome as a gene presence/absence matrix. In practice, clusters are generated first based on sequence similarity, with paralogs being identified using either synteny-based (Page et al. 2015; Tonkin-Hill et al. 2020) or tree-based approaches (Ding et al. 2018; Zhou et al. 2020). Roary (Page et al. 2015), developed in the first generation of these tools, generates COGs based on a single BLAST threshold without correction for gene prediction errors. Later tools introduced lower identity thresholds to better cluster divergent gene families, with additional processing to reduce the effects of gene prediction and annotation errors. Panaroo (Tonkin-Hill et al. 2020) uses synteny and population-frequency information to identify spurious COGs originating from contaminants or fragmentation, corrects out-of-frame errors in which fragmentation results in incorrect frame prediction, and predicts genes that may have been missed initially by gene-callers. Panaroo also corrects annotation errors by only keeping the best-supported annotation within a COG. However, Panaroo focuses on producing accurate COGs and does not directly correct gene predictions. Its reliance on gene synteny for clustering correction can also limit its ability to deal with highly fragmented assemblies. PEPPAN (Zhou et al. 2020) addresses prediction errors by generating COGs initially, before identifying the longest sequence for each COG and searching for its homologs within all genomes in the data set. This process ensures that all gene start and stop coordinates within a COG are predicted consistently. However, PEPPAN does not use the same stringent quality-control methods used in Panaroo, making it susceptible to errors originating from low-quality assemblies. Both Panaroo and PEPPAN also rely on gene prediction and annotation within individual genomes, which is computationally inefficient. There is currently no tool that corrects for poor assembly quality and gene prediction errors and avoids redundancy in gene prediction and annotation.

To enable nonredundant, consistent, and accurate gene prediction and annotation across a population, a data structure is required that represents the distribution of genetic variation across many genomes. Pangenome graphs provide a means of compacting large collections of linear references into a network, in which identical or similar sequences are merged into nodes, variation is represented by edges, and individual genomes are represented as paths through the graph (Eizenga et al. 2020). De Bruijn graphs (DBGs) are a form of pangenome graph that are built from matching short nucleotide sequences known as *k-*mers, with edges added between *k-*mers that share an overlap of *k*−1 nucleotides. Colored compacted DBGs compress nonbranching paths of *k-*mers into sequences called “unitigs,” with each *k-*mer being annotated with the genomes, or “colors,” in which it is found. DBGs are a highly scalable method of building pangenome graphs, capable of including thousands of bacterial genomes (Holley and Melsted 2020), and provide a lossless representation of population diversity (Schulz et al. 2022). DBGs therefore do not have the redundancy of the equivalent collection of linear genomes and have the potential to consistently predict and annotate genes, informed by node-level population frequency. This functionality is available in Pantools, which generates consistent and nonredundant functional ortholog annotation of genes on DBGs (Jonkheer et al. 2022). However, prior gene prediction in linear genomes is still required. Gene prediction and annotation within a DBG would therefore overcome the issues encountered when conducting pangenome analysis using individual linear genomes.

Here, we present ggCaller, a population-wide gene-caller based on DBGs. ggCaller uses population-frequency information to guide gene prediction, aiding the identification of homologous start codons across orthologs, as well as consistent scoring and functional annotation of orthologs. ggCaller also includes a query mode, enabling reference-agnostic functional inference for sequences of interest, applicable in pangenome-wide association studies (PGWAS). We show the accuracy and computational benefits of graph-based gene prediction and annotation using simulated and real bacterial genomes, comparing ggCaller to existing state-of-the-art tools.

## Results

### Overview of the ggCaller workflow

**Figure 1.**
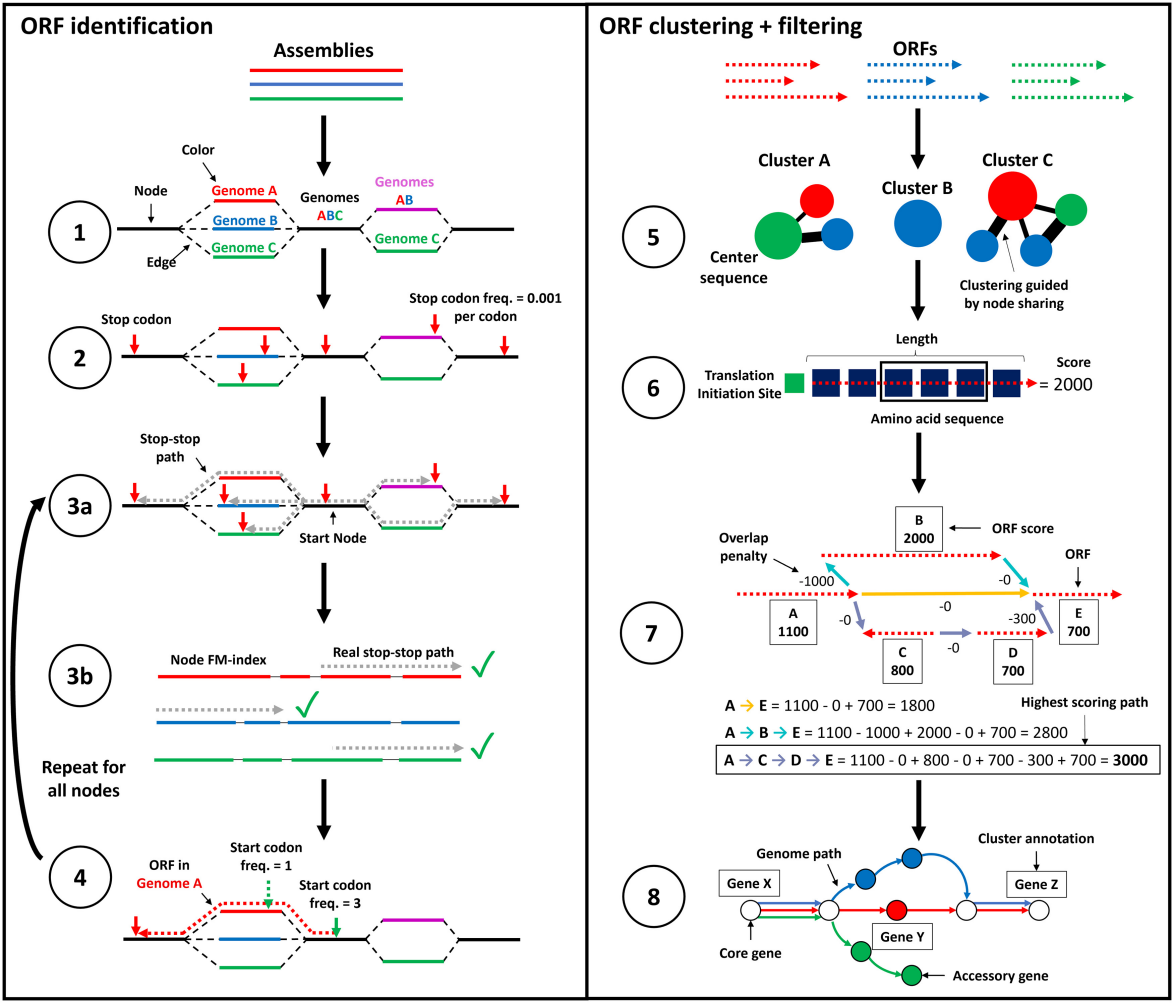
ggCaller workflow. ggCaller can be split into two sections: ORF identification (steps 1–4) and ORF clustering + filtering (steps 5–8). (1) DBG is generated from assemblies by Bifrost. (2) All stop codons are identified, and stop frequency is calculated (total number of stop codons in DBG / total number of codons in DBG). (3a) Starting at an initial node containing a stop codon, a depth first search (DFS) is used to pair all stop codons in the start node with a downstream stop codon in the same reading frame. (3b) During DFS, paths are compared to an FM-index to remove incorrect paths. (4) ORFs are defined by identifying start codons scored based on translation initiation site sequence, genome coverage (given by number of colors shared in node), and frequency of this start being chosen in other potential orthologs. Steps 3 and 4 are repeated for all nodes containing a stop codon. (5) ORFs are clustered into COGs, using node-sharing to reduce search space. (6) Balrog is used to generate an average per-residue score using only the center sequence of each COG. This average per-residue is used to score each ORF in the center sequence's respective cluster. (7) Highest scoring tiling path calculated for overlapping genes within the DBG using the Bellman–Ford algorithm (Bellman 1958; Ford and Fulkerson 1962), producing a “true” gene-call set. (8) Gene-calls and synteny information are used to build a gene graph. A modified version of Panaroo is used to remove poorly supported gene-calls, annotate clusters, and recall missed genes/pseudogenes.

ggCaller predicts genes within a DBG (Fig. 1), using sequence sharing across the whole population to guide prediction, clustering, and annotation of orthologous genes (a detailed overview of the ggCaller workflow can be found in the Supplemental Methods). DBGs are generated by Bifrost (Holley and Melsted 2020) from assemblies in FASTA format (step 1). We chose Bifrost because of its scalability and comprehensive representation of variation at small and large scales (Andreace et al. 2023). DBGs are constructed by first matching *k-*mers (sequences of length *k*, which is chosen a priori by the user). Nonbranching paths of *k-*mers are merged into unitigs (from here referred to as “nodes”). These nodes are “colored” based on the input genomes they are found in, enabling calculation of the population frequency of all sequences greater than *k* bases in length.

ggCaller then identifies all stop codons in the DBG (step 2) and traverses the DBG to identify putative gene sequences, known as open reading frames (ORFs) (step 3). Each stop codon is paired with a downstream stop-codon in the same reading frame using a depth first search (DFS) (step 3a), thereby delineating the coordinates of all possible reading frames. To remove artifacts generated by “short-circuits” in the DBG (Břinda et al. 2021), candidate paths are searched against the contiguous input sequences using an FM-index rank query (step 3b).

The sequences between pairs of stop codons are then searched for start codons, which are paired with downstream stop codons in the same reading frame, generating an ORF. As bacterial genes have alternative start sites due to reuse of start codons within an exon (Dimonaco et al. 2022), the best supported start site is chosen based on their sequence and respective population frequency (step 4, see Supplemental Methods).

ORFs are then clustered using a method based on Linclust (Steinegger and Söding 2018). ORFs are compared only to “center sequences” (step 5), which are the longest ORFs with which they share a common node, rather than exhaustively against all other ORFs. Edlib (Šošić and Šikić 2017) is used to rapidly calculate pairwise edit distances in amino-acid space. ORFs are then clustered with the center sequence with which they share the highest identity.

ORFs are then scored using the gene sequence-scoring model in Balrog (Sommer and Salzberg 2021), a temporal convolutional network that generates an average per-residue score for a translated ORF sequence (step 6). Scores are generated only for center sequences, which are then applied to the remaining ORFs in their respective clusters, vastly reducing the number of Balrog model queries required to score all ORFs. ORF scores are then used to determine the highest-scoring tiling path through the DBG per input genome, which penalizes large overlaps between adjacent ORFs (step 7). This generates a population-wide set of CDS predictions.

CDS predictions are then passed to an updated version of Panaroo's gene graph algorithm (Tonkin-Hill et al. 2020) that has been adapted to work directly with DBGs rather than linear genomes. CDSs are clustered further down to 50% identity, paralogous CDS clusters are split, and poorly supported clusters are removed (step 8). This step generates a graph with nodes representing COGs, rather than DNA sequences as used in the Bifrost DBG. ggCaller uses the same three presets for COG pruning as implemented in Panaroo: sensitive, moderate, and strict (see Supplemental Methods), generating clusters containing final gene-calls. Clusters are also functionally annotated using DIAMOND (Buchfink et al. 2015) and/or HMMER3 (Eddy 2009). As in step 6, only cluster center sequences are queried, with the functional annotation being shared across all genes in the cluster. ggCaller also implements a DBG-based gene refinding module, which enables recalling of genes or pseudogenes missed on the first pass by ggCaller. The final default outputs are a gene presence/absence matrix, a set of annotated gene clusters and their respective sequences, and their locations in their respective linear input sequences (as standardized GFF3 files). Additionally, core/pangenome alignments, phylogenies, and single-nucleotide polymorphism calls can be generated automatically.

ggCaller features several innovations over existing gene annotation and pangenome analysis tools. Steps 2–4 ensure start positions of orthologs are called consistently across a population by considering population frequencies of start codons. This process was implemented to avoid incorrect ORF truncation or extension, which is an issue with one-by-one linear-genome gene-calling. Steps 5 and 6 reduce the number of Balrog model queries and ensure orthologs are scored equally. Only scores for cluster center sequences are generated, which can then be shared across orthologs that have the same or similar scores due to sequence similarity. Similarly, in step 8, ggCaller functionally annotates clusters using only center sequences. Both processes were designed to reduce annotation inconsistency and redundancy across orthologs to increase gene prediction and annotation accuracy and lower runtime.

### ggCaller accurately predicts genes in incompletely assembled structurally diverse operons

To initially benchmark gene prediction accuracy from ggCaller, we predicted genes in a collection of five pneumococcal capsular polysaccharide biosynthetic operons (*cps*) (Bentley et al. 2006), comparing predictions with the previously annotated gene coordinates. These *cps* operons were chosen as they are highly diverse in sequence content and structure, consisting of between 16 and 23 genes, and are manually curated, providing an ideal initial ground-truth data set. To simulate the assembly errors seen in draft assemblies, analysis was conducted on fully intact *cps* sequences and on sequences in which all manually curated genes were synthetically fragmented with a single contig break at a random position. Genes were predicted with ggCaller in moderate mode and with GeneMarkS-2 and Prokka (which uses Prodigal for gene prediction) using Panaroo in moderate mode. As all workflows used Panaroo for pangenome analysis, results are henceforth referred to only by the respective gene-prediction tool. We compared tools based on their recall and precision of the ground-truth gene set, and on the length of these sequences covered by the respective predictions for each gene-prediction tool, to determine how predictions were affected by fragmentation.

**Figure 2.**
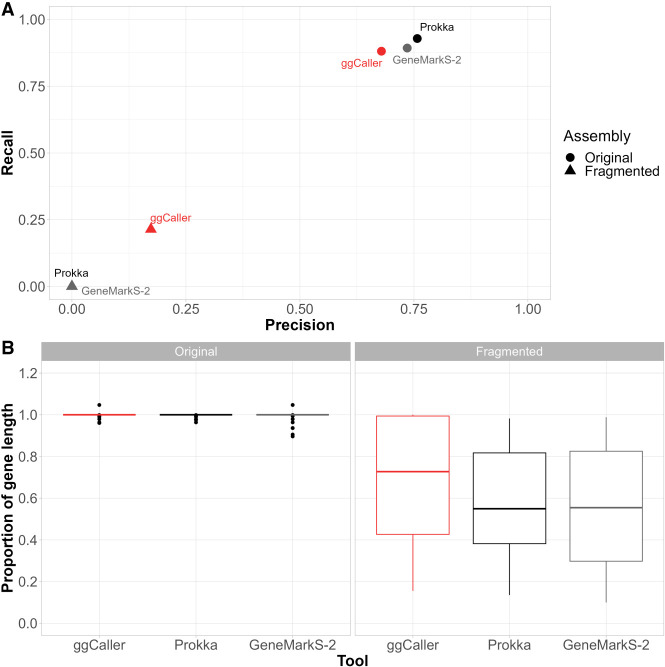
Gene prediction comparison for pneumococcal capsular biosynthetic operons with and without simulated fragmentation. (*A*) Precision versus recall comparisons for correctly identified genes (i.e., correct start and end coordinates) and (*B*) proportion of ground-truth gene length included in prediction if 3′ end is correctly called for original and fragmented ground-truth genes within *cps* operons.

The recall and precision of exact matches to ground-truth gene sequences were compared across the original and fragmented *cps* operons (Fig. 2A). ggCaller performed similarly to other tools in terms of recall in the original *cps* operons, albeit with slightly lower precision. To determine why precision was slightly lower, we compared the 3′ accuracy of predictions, whereby the 3′ end of a prediction matches a ground-truth sequence; however, the 5′ end may over- or undershoot that of the ground truth. All tools recalled all genes correctly, whereas ggCaller precision was still slightly lower than the other tools (Supplemental Fig. 1; Supplemental Table 1). Analysis of false-positive lengths showed that those shared between ggCaller and other tools were the same length (Supplemental Fig. 2), and those exclusive to each workflow were shorter when there was no 3′ match to ground-truth sequences (Supplemental Fig. 3). Therefore, small differences in accuracy between ggCaller and other tools were due to variations in 5′ identification across genes, as well as in identification of short ORFs (<500 bp), which are notably difficult to predict (Dimonaco et al. 2022).

In fragmented *cps* operons, ggCaller was the only tool able to recall any genes with correct start and end coordinates, highlighting reduced sensitivity to assembly fragmentation over linear-genome gene-prediction tools. Tools were also compared based on the proportion of the curated gene length covered by their respective gene-calls (Fig. 2B). In the original *cps* operons, most gene predictions from all tools fully covered their respective ground-truth sequence. However, in the fragmented *cps* operons, ground-truth sequences were covered to a greater degree by ggCaller predictions (median: 0.68) than by Prokka or GeneMarkS-2 (median: 0.57, 0.55, respectively). As Bifrost DBGs connect *k-*mers with a *k* − 1 overlap anywhere in the population (Holley and Melsted 2020), contig breaks in individual assemblies can be spanned by forming a path across *k-*mers in other assemblies that do not have contig breaks at that orthologous position. This enables ggCaller to recall a greater number of full gene sequences in highly fragmented assemblies than linear-genome gene-callers.

### ggCaller has superior performance in simulated data sets with complex sources of assembly error

In addition to gene-prediction, ggCaller provides a single workflow for annotation, ortholog clustering, and pangenome analysis. To benchmark ggCaller against existing pangenome analysis workflows, we generated simulated populations of 100 assemblies starting from *Streptococcus pneumoniae* ATCC 700669 serotype 23F (referred to as “Spn23F”) using the workflow described in Tonkin-Hill et al. (2020). We chose to use simulations over transcriptome-based gene predictions to provide ground-truth gene sets, as transcriptomics is not the standard for bacterial gene prediction and may not capture all functional sequences (Salzberg 2019). Briefly, we simulated populations containing 100 genomes, using varying gene gain/loss ratios and within-gene mutation rates, as well as additional fragmentation or contamination with fragments of *Staphylococcus epidermidis*, a common contaminant (see Methods). This resulted in seven separate parameter combinations. To more accurately simulate the real-world processes involved in pangenome analysis, assemblies were generated from simulated genomes using ART to generate error-prone reads, and SPAdes to assemble these (Bankevich et al. 2012; Huang et al. 2012). ggCaller was compared against three workflows: genes were first identified and annotated by Prokka, and pangenome analysis was conducted either using either Roary, Panaroo, or PEPPAN (each workflow further referred to only by the pangenome analysis tool). We then compared the workflows based on their estimates of total pangenome size, accessory genome size (defined as COGs present in <99% of genomes), and core genome size (defined as COGs present in ≥99% of genomes) compared to the expected number of COGs provided by the simulation to determine pangenome representation accuracy.

**Figure 3.**
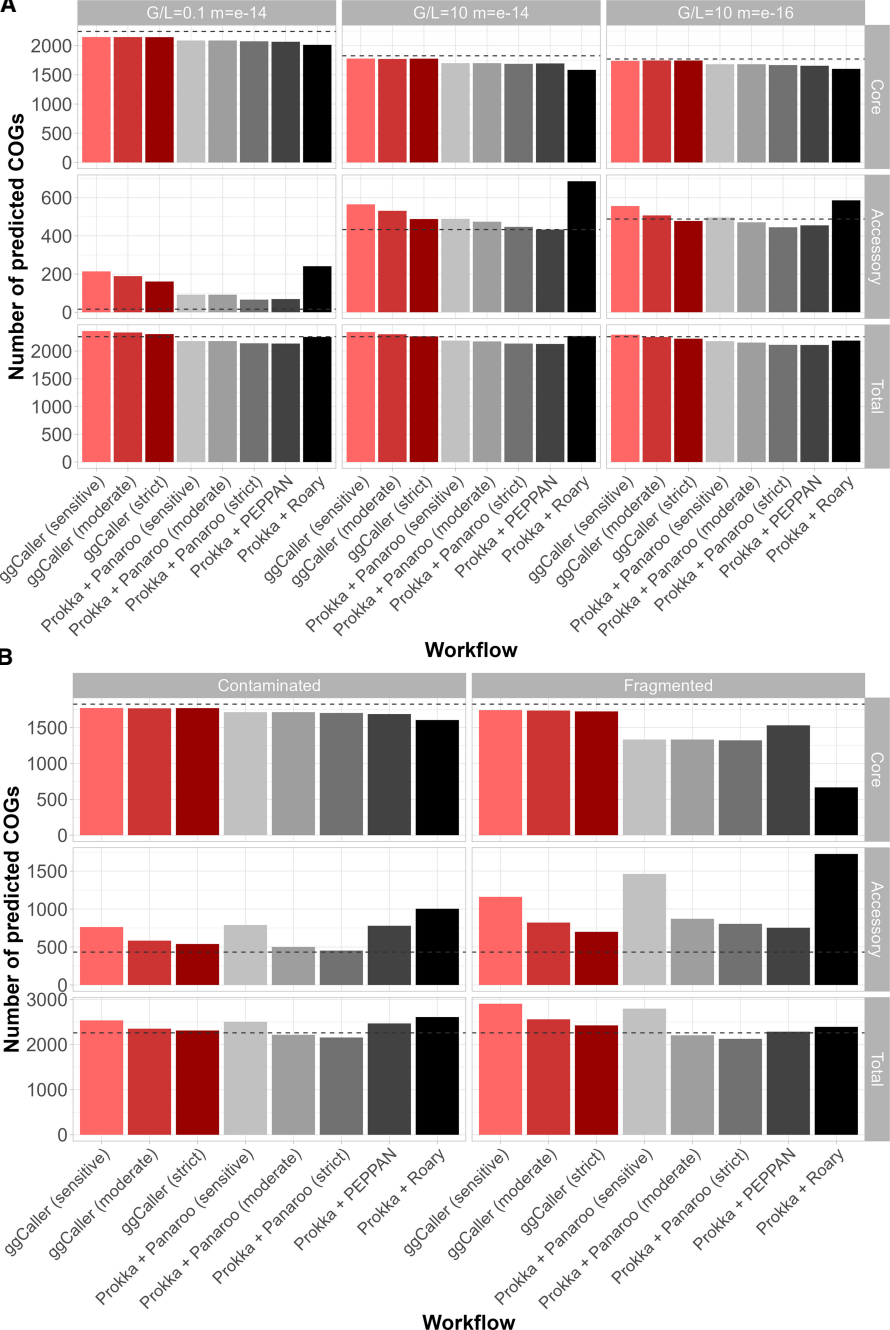
Comparison of estimated core, accessory, and total pangenome sizes across simulated populations. Panels describe simulations with simple (*A*) and complex (*B*) sources of error. Bars indicate the predicted number of COGs for each workflow. Ground-truth values are represented by the gray dotted line in each panel. *Horizontal* panels describe simulation parameters; *vertical* panels describe COG frequency: core (99% ≤x ≤ 100%; *top*), accessory (0 ≤x < 99%; *middle*), and total (0 ≤x ≤ 100%; *bottom*).

Comparisons of estimated core and accessory genome and total pangenomes sizes are shown in Figure 3 (for data, see Supplemental Table 2). Simulations were split into simple (Fig. 3A) and complex (Fig. 3B); simple simulations varied based on gene gain/loss ratio (denoted as “G/L”) and per-site mutation rate (denoted as “m”, in mutations per gene per genome per generation), and complex simulations were fragmented or contaminated with fragments of *S. epidermidis* (for results for simulations not shown above, see Supplemental Fig. 4). For all simulations, ggCaller estimated the largest core genome and was closest to the ground-truth of all tools, independent of stringency settings. Differences in core genome size were particularly notable in the fragmented simulation, with ggCaller predicting ≥194 more core COGs than PEPPAN across stringency modes, the next best-performing workflow. As highlighted in Figure 2, ggCaller can recall a greater number of intact genes in highly fragmented assemblies, which improves clustering accuracy by generating fewer truncated orthologs. In contrast, Panaroo, PEPPAN and Roary all underestimated core genome size to a greater degree and overestimated accessory genome size. The greatest difference was seen in Panaroo and Roary, highlighting an issue in clustering when many assemblies in the data set are highly fragmented. As Panaroo and Roary rely on gene synteny to guide clustering, when this is incorrect or inconsistent in the input (Page et al. 2015; Tonkin-Hill et al. 2020), underclustering of COGs can occur. PEPPAN was less sensitive to fragmentation due to the use of gene trees in addition to gene synteny to generate COGs, reducing the effect of assembly fragmentation (Zhou et al. 2020); however, it was still less accurate than ggCaller.

Estimations of accessory genome sizes were more varied across all tools, with strict modes in ggCaller or Panaroo recapitulating the ground-truth most accurately across a majority of simulations. In simple simulations, ggCaller overestimated accessory genome size in simulations with high mutation rate (m = 10^−14^) and was outperformed by Panaroo and PEPPAN, although estimates were still lower than Roary. However, in the simple simulation with lower mutation rate (m = 10^−16^), ggCaller (strict) was only two COGs less accurate than Panaroo (sensitive), which was closest to the ground-truth. In complex simulations, ggCaller (strict) estimates of accessory genome size were third to Panaroo in moderate and strict modes in the contaminated simulation and were the most accurate in the fragmented simulation. Therefore, ggCaller accessory genome estimation accuracy is more variable than for the core genome. However, performance is often similar to, or better than, existing gold-standard tools.

**Figure 4.**
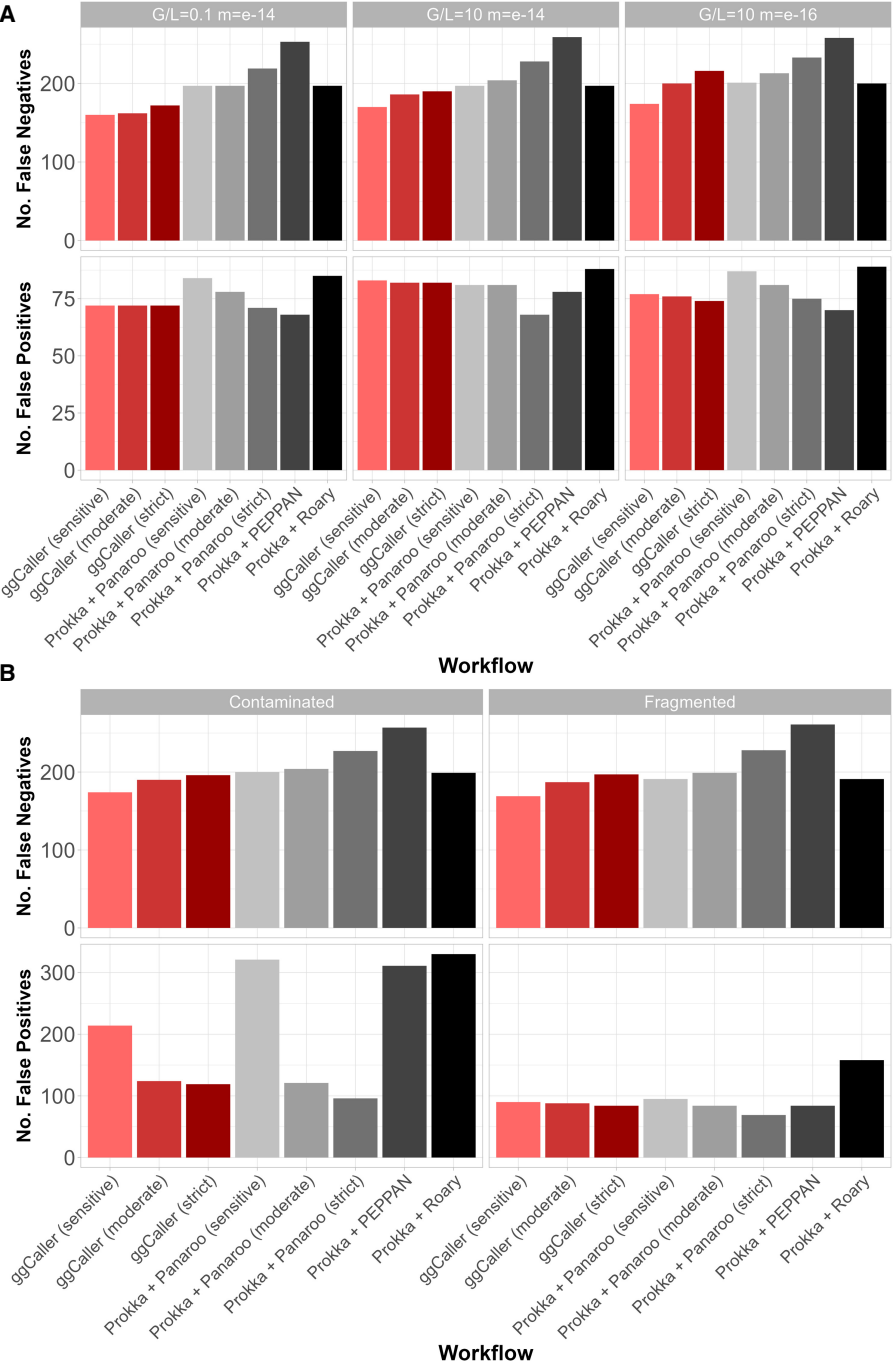
Comparison of COG annotation accuracy across simulated populations. Panels describe simulations with simple (*A*) and complex (*B*) sources of error. False negatives are COGs that were present in the ground-truth set but not called by a workflow. False positives are COGs that were called by a workflow but were not present in the ground-truth set.

The recall and precision of expected COGs were also compared to quantify differences in gene annotation and clustering accuracy in simple (Fig. 4A) and complex (Fig. 4B) simulations (for data, see Supplemental Table 3; for results for simulations not shown above, see Supplemental Fig. 5). Notably, ggCaller in sensitive and moderate modes had the fewest false negatives across all simulations, in line with ggCaller's more accurate core genome size estimation (Fig. 3). For false positives, ggCaller (strict) was second only to Panaroo (strict) or PEPPAN. PEPPAN and Roary had the highest number of false positives in the contaminated simulation (311 and 330, respectively), whereas ggCaller and Panaroo performed similarly, ranging between 119 and 214 and between 96 and 321 for varying stringency, respectively. Within correctly predicted COGs, ggCaller had similar numbers of errors to those of Panaroo and PEPPAN, with Roary performing worst (Supplemental Fig. 6). Overall, these simulations show that ggCaller performs as well as, or better than, gold-standard pangenomic analysis workflows in simulated populations, particularly when estimating core genome size.

### ggCaller accurately represents pangenomes of bacterial species with varying levels of diversity

To benchmark ggCaller on real-world data, we analyzed genome sequences from three bacterial species with varying patterns of pangenome diversity. *Mycobacterium tuberculosis* is a slow-replicating respiratory pathogen with a low mutation rate and a small accessory genome (approximately 4000 core genes, fewer than 1000 accessory genes) (Yang et al. 2018). *S. pneumoniae* is a nasopharyngeal commensal and pathogen that exchanges genetic material through homologous recombination and has a relatively small core and large accessory genome (approximately 1000 core genes, more than 5000 accessory genes) (Corander et al. 2017). *Escherichia coli* is a genetically diverse enteric bacterium, with an intermediate-size core genome, but extensive accessory genome (approximately 3000 core genes, more than 100,000 accessory genes) (Park et al. 2019). These three species are important, commonly studied pathogens that represent a broad range of pangenome diversity, providing a diverse benchmarking data set. Genomes for *M. tuberculosis* (N = 219), *S. pneumoniae* (N = 616), and *E. coli* (N = 162) were collated from public repositories (see Methods). Although these data sets are relatively small by modern standards, the genomes are representative of the observed diversity within each species (Lees et al. 2019) and are suitable for identifying differences in performance between tools. The same workflows as above were compared based on respective predicted COG frequencies.

**Figure 5.**
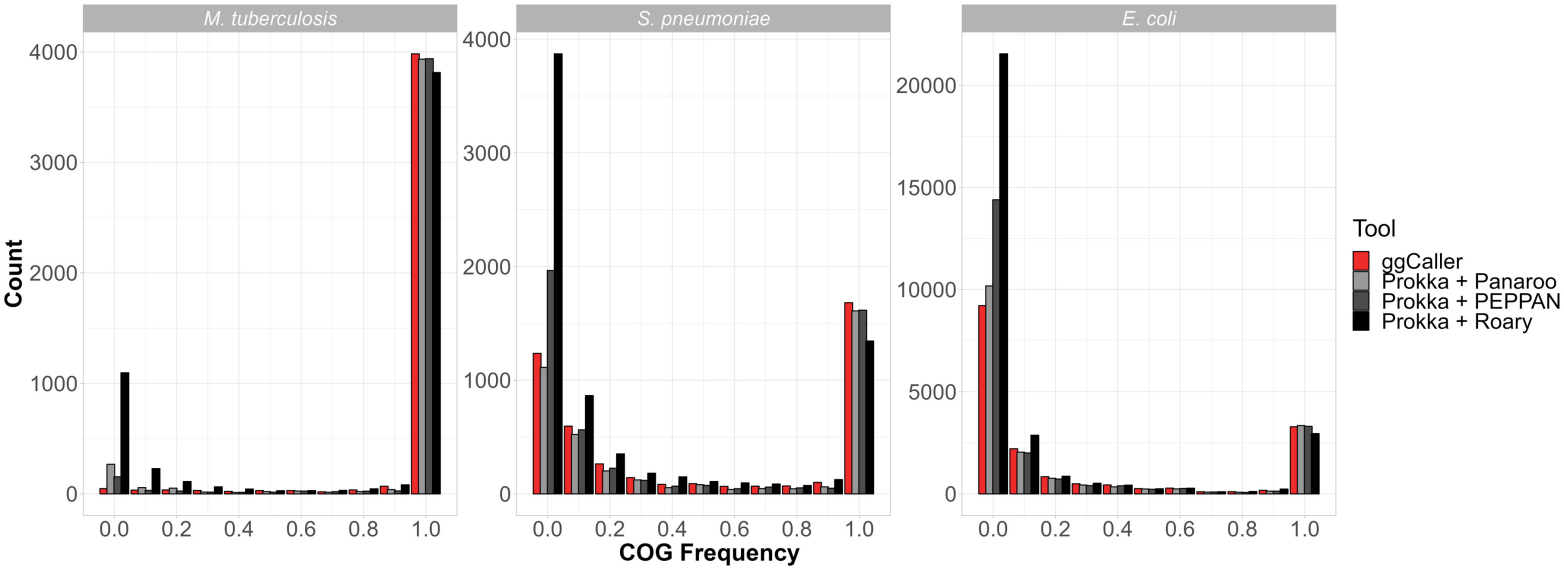
Gene frequency histograms for *Mycobacterium tuberculosis*, *Streptococcus pneumoniae*, and *Escherichia coli* across pangenome analysis workflows. ggCaller and Panaroo were run in strict mode.

Predicted gene frequency histograms for each species and workflow are shown in Figure 5, with counts of COGs with 100% frequency, <100% frequency, and total COGs in each pangenome in Table 1. For *M. tuberculosis*, all tools predicted more than 3800 COGs at between 90% and 100% frequency, with ggCaller predicting the most in this frequency bin (3982). ggCaller and Panaroo predicted similar numbers at 100% frequency (3679 and 3680, respectively), and PEPPAN predicted the most (3726). All tools, except for Roary, predicted a minimal accessory genome, with ggCaller predicting the fewest COGs in the lowest gene frequency bin (48, 0%–10% frequency). This result is consistent with previous analysis using Panaroo (Tonkin-Hill et al. 2020). Notably, Roary predicted the highest number of COGs in the lowest bin (1096, 0%–10% frequency) and the highest number of total COGs, likely owing to its strict clustering threshold.

**Table 1.**
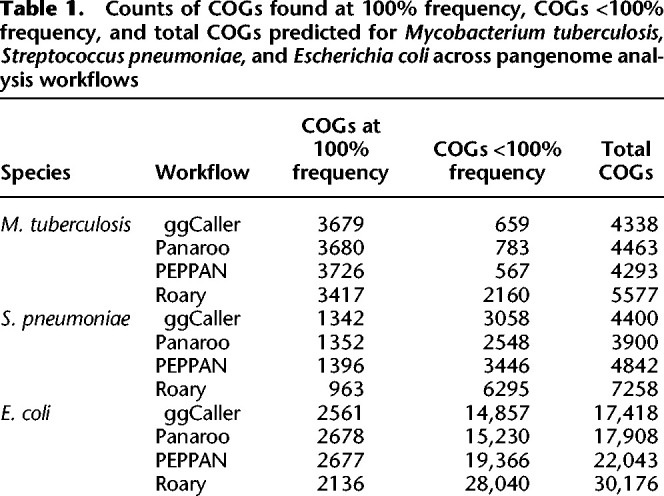
Counts of COGs found at 100% frequency, COGs <100% frequency, and total COGs predicted for *Mycobacterium tuberculosis*, *Streptococcus pneumoniae*, and *Escherichia coli* across pangenome analysis workflows

For *S. pneumoniae*, ggCaller, Panaroo, and PEPPAN predicted 1682, 1610, and 1616 COGs at between 90% and 100% frequency, respectively, whereas Roary predicted 1345 COGs for the same bin. For the same data set, Croucher et al. (2013a) predicted 1194 COGs at 100% frequency and 5442 total COGs. All tools except for Roary predicted a higher number of COGs found at 100% frequency and lower total COGs. These differences are likely due to more accurate ortholog clustering compared with the original study, which used a combination of manual steps and COGsoft (Kristensen et al. 2010). More accurate clustering would both reduce the total number of unique COGs reported and increase the number of high frequency COGs, as seen here for ggCaller, Panaroo, and PEPPAN. For low-frequency COGs, ggCaller and Panaroo estimated a similar number between 0% and 10% frequency (1236 and 1113, respectively), with PEPPAN predicting a greater number (1965). Again, Roary estimated the largest number of COGs at 0%–10% frequency (3871) and estimated the largest number of total COGs of all workflows (7258).

For *E. coli*, ggCaller, Panaroo, PEPPAN, and Roary estimated a similar number of genes with frequencies in the range of 90%–100% (3287, 3308, 3345, and 2953 respectively). Estimates of the *E. coli* core genome vary depending on the composition and size of the data set being analyzed and have previously been reported to be in the range of 800–3000 COGs (Chen et al. 2006; Kallonen et al. 2017; Park et al. 2019). Therefore, all predictions were within the expected range for *E. coli*, despite Roary predicting approximately 300 fewer COGs than the other workflows. ggCaller predictions were consistent with Panaroo and PEPPAN for all frequency compartments, except for COGs found at 0%–10% frequency, for which PEPPAN predictions were elevated (9216, 10,181, and 14,399 for ggCaller, Panaroo, and PEPPAN, respectively), as seen before with *S. pneumoniae*. This was also consistent with previous simulation results with PEPPAN (Fig. 4B).

To determine the consistency of gene predictions within COGs, we compared the coefficient of variation (CV) of gene lengths and number of genes per COG across workflows (Supplemental Fig. 7). Smaller CVs, coupled with larger numbers of genes within COGs, indicate greater consistency in gene predictions and clustering. Comparisons of within-COG length CV highlighted that ggCaller COGs were less variable than Panaroo and Roary in terms of gene lengths on average for all species, with PEPPAN having lowest variation. Contrastingly, the number of genes per COG varied by workflow and species. For *M. tuberculosis*, ggCaller COGs contained more genes than Roary, although they contained fewer genes than Panaroo, whereas PEPPAN generated COGs containing the largest number of genes on average. For *S. pneumoniae*, ggCaller again identified COGs containing fewer genes than Panaroo; however, they contained more genes than those of PEPPAN and Roary on average. For *E. coli*, ggCaller and Panaroo performed similarly, with COGs containing more genes than PEPPAN and Roary on average. Therefore, although ggCaller lowers CV by a smaller degree than PEPPAN, PEPPAN generated smaller COGs in more diverse bacterial species (*S. pneumoniae* and *E. coli*), which was not observed with ggCaller.

In this analysis of real bacterial populations, ggCaller performed equivalently to, or better than, existing gold-standard pangenome analysis tools across a broad range of bacterial species and provided gene frequency predictions in line with previous studies.

### ggCaller annotates structurally complex and repetitive genes more accurately than existing tools

Previous analysis in simulated populations highlighted that increased within-gene divergence can impact estimates of pangenome size and COG annotation (Fig. 3A). Therefore, genes may be inaccurately annotated or clustered by current approaches owing to sequence or structural diversity, for example, in antigens under diversifying selection (Croucher et al. 2017). To determine the effect of allelic and structural variation within genes on clustering accuracy in ggCaller and alternative tools, we compared examples of structurally diverse COGs from the *S. pneumoniae* data set used previously. Four structurally diverse proteins were chosen: penicillin binding proteins 1a and 2b (Pbp1a, Pbp2b), Pneumococcal surface protein A (PspA), and Pneumococcal serine-rich repeat protein (PsrP). Pbp1a and Pbp2b are clinically important due to conferral of beta-lactam resistance and vary structurally through interspecies recombinations, generating mosaic sequences (Croucher et al. 2013a). PspA is an important virulence factor under positive selection by the immune system, which has generated wide structural diversity. Finally, the presence of repeats in PsrP (more than 1000 repeats of the SASX motif) (Shivshankar et al. 2009) presents a particular challenge for assemblers and gene prediction tools (Croucher et al. 2017).

**Figure 6.**
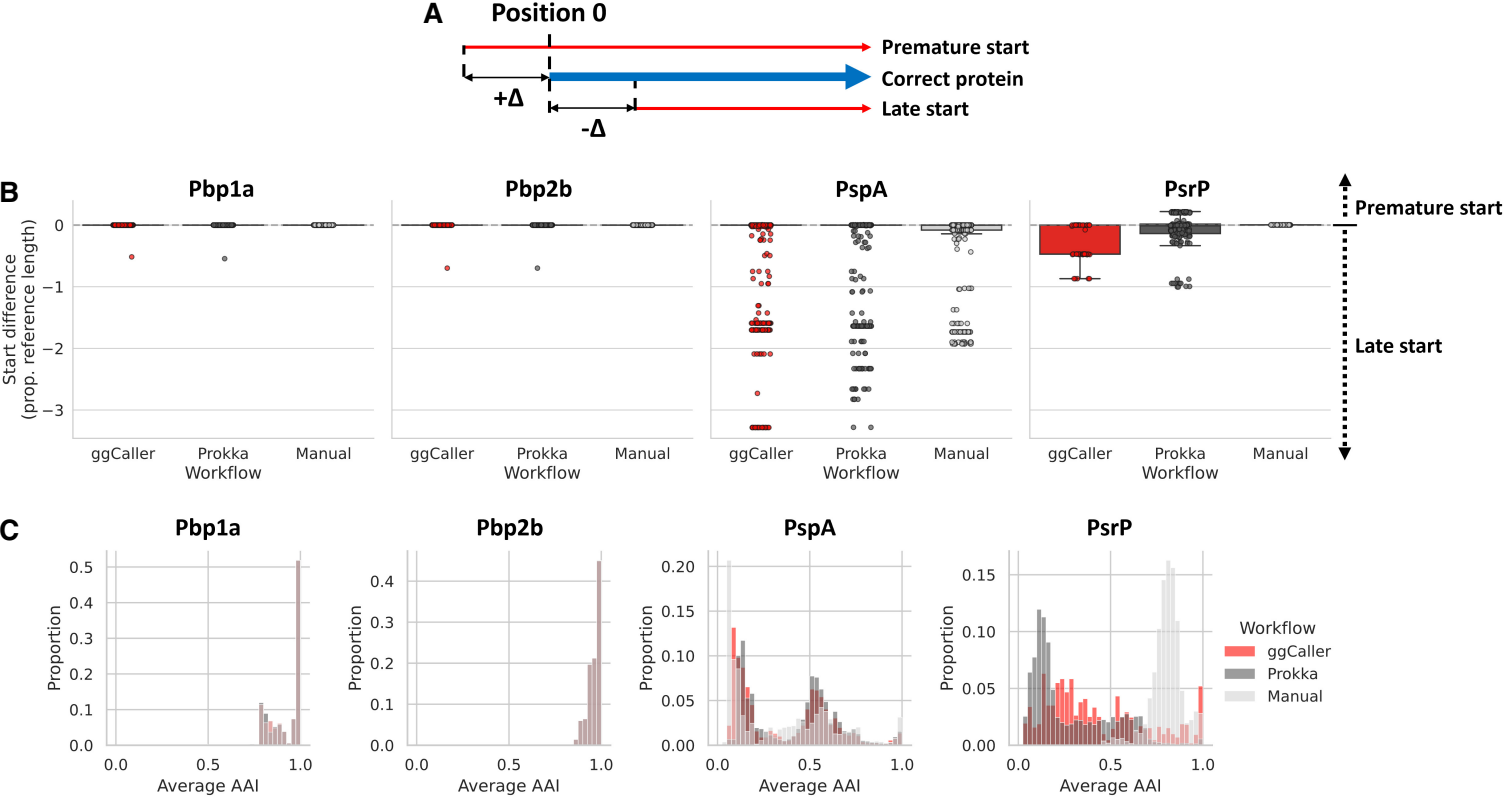
Comparison of within-COG start site soft-clipping and average amino acid identity (AAI) between gene prediction workflows. (*A*) Description of start site soft-clipping. (*B*) Boxplot comparisons of start site soft-clipping protein sequences of Pbp1a, Pbp2a, PspA, and PsrP based on alignment with the manually annotated reference in Spn23F. (*C*) Histograms of pairwise average AAI within each COG.

To benchmark the annotation and clustering accuracy of these genes, we compared gene prediction and pangenome analysis workflows based on the consistency of predicted start and stop coordinates, sequence identity, and the total number of sequences within each COG. As a benchmark, predictions were also compared to the original predicted protein sequences from Croucher et al. (2015), in which genes were predicted using multiple gene-callers followed by manual inspection to increase accuracy and were clustered using COGsoft (referred to as “Manual + COGsoft”) (Kristensen et al. 2010). Protein sequences from predicted genes were aligned to manually curated reference sequences from Spn23F. Differences between the start and stop positions were compared using the number of amino-acids soft-clipped at either end of the alignment to Spn23F. Soft-clipping is a measure of the “overhang” of an alignment, with bases in a soft-clip not being aligned to amino acids in the other sequence. Here, a positive value means the query sequence is soft-clipped; a negative value means the correct sequence is soft-clipped; and a value of zero means perfect alignment (Fig. 6A). Pairwise average amino acid identity (AAI) was also calculated for all sequences within each COG (proportion of matching amino acids over the gapped alignment length) (Doolittle 1981; Raghava and Barton 2006). Comparing distributions of average AAI across tools provides a measure of clustering accuracy; peaks at low average AAI indicate that start or stop sites are inconsistently predicted between orthologs, whereas peaks at high AAI suggest good consistency in gene prediction within a COG. The numbers of sequences within each COG were also considered, to ensure that the absence of low average AAI peaks was not a consequence of COGs containing fewer sequences.

Comparisons of start site soft-clipping between ggCaller, Prokka, and the gene prediction data from the original study (Manual) are shown in Figure 6B, and distributions of pairwise average AAI are shown in Figure 6C. For Pbp1a and Pbp2b, almost all predicted proteins matched the start positions within the Spn23F reference for ggCaller and Prokka and were consistent with Manual predictions. Both workflows also had equivalent distributions of AAI and matched Manual predictions with modal peaks at 1.0, indicating consistent prediction and clustering of orthologs. The number of Pbp1a and Pbp2b orthologs, and the genomes they were found in, also matched between ggCaller and Manual + COGsoft; however, Prokka + Panaroo missed a single isolate containing Pbp2b (Table 2).

**Table 2.**
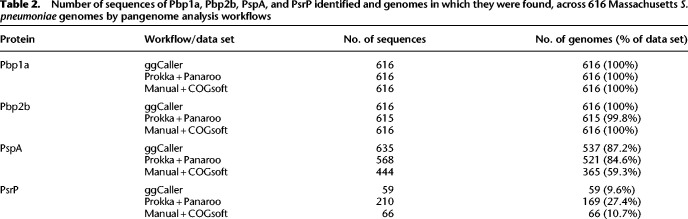
Number of sequences of Pbp1a, Pbp2b, PspA, and PsrP identified and genomes in which they were found, across 616 Massachusetts *S. pneumoniae* genomes by pangenome analysis workflows

For PspA, distributions of start site variants, as well as AAI distributions, were consistent between ggCaller and Prokka but were variable within the respective COGs. Based on raw FASTA files (see Supplemental Code), predictions were affected by assembly fragmentation, causing truncation of sequences with ggCaller and Prokka in some cases, resulting in differences in start and stop codon position, although a majority of positions matched the reference (Fig. 6B; Supplemental Fig. 8). Therefore, ggCaller and Prokka identified similar levels of diversity in PspA. For Manual predictions, there was a greater proportion of truncated proteins present than both the other workflows, and although its respective AAI distribution was largely consistent with ggCaller and Prokka + Panaroo, fewer PspA orthologs were identified. PspA is a core gene in *S. pneumoniae* (Croucher et al. 2017) and therefore should be identifiable in all isolates. ggCaller had greater recall than the Prokka + Panaroo and Manual + COGsoft workflows, identifying PspA in 16 and 172 more isolates, respectively.

For PsrP, ggCaller gene annotations had three distinct truncated starting positions, whereas Prokka predictions were more variable, and Manual had no variation. Ranges of start site positions between ggCaller and Prokka were similar and were a result of fragmentation in assemblies causing truncated predictions (see Supplemental Code), as before with PspA. ggCaller had a broad modal peak at ∼0.25 AAI, whereas Prokka and Manual had peaks at about 0.1 and about 0.8 AAI, respectively. Notably, ggCaller had the highest proportion of exact AAI matches (AAI = 1.0), indicating greater consistency in PsrP predictions than other workflows. PsrP stop site predictions with Manual predictions were almost all truncated, whereas most ggCaller and Prokka predictions matched Spn23F (Supplemental Fig. 8). This discrepancy explains the higher modal peak at about 0.8 AAI for Manual predictions; fewer repeat units were included in PsrP sequences, leading to more closely matching sequences, whereas ggCaller correctly predicted more gene end coordinates. From the raw FASTA files (see Supplemental Code), only 15/210 PsrP sequences contained an SASX motif for Prokka, compared with 54/59 and 66/66 for ggCaller and Manual predictions, respectively. The DAE motif, present in incorrect CDSs translated from the antisense strand within *psrP*, was found in 148/210 sequences identified by Prokka, whereas it was not found in any for ggCaller or Manual predictions. ggCaller was also consistent with Manual + COGsoft in terms of the number of genes within the COG, which identified PsrP in 59 and 66 genomes, respectively, versus 169 in Prokka + Panaroo. The underestimation in genes per COG by ggCaller compared with Manual + COGsoft is likely owing to CDSs being fragmented across contig breaks, meaning ggCaller was not able to successfully pair a start and stop codon. For Prokka + Panaroo, the poor alignment of start sites and modal peaks at low AAI indicate inflation of the PsrP COG owing to clustering of incorrectly predicted CDSs by Prokka. Overall, ggCaller outperformed a workflow of Prokka + Panaroo when clustering structurally diverse proteins.

### ggCaller improves functional interpretation in pangenome-wide association studies

ggCaller supports querying of sequences of arbitrary length within an annotated DBG, enabling reference-free functional interpretation of sequence elements. This is useful when analyzing significant hits from a PGWAS, in which current approaches annotate results by mapping to only one or a few references (Lees et al. 2018). To show the utility of ggCaller for this purpose, we performed PGWAS to identify sequences significantly associated with tetracycline and macrolide resistance in *S. pneumoniae.* Tetracycline resistance is caused by presence of *tetM* in *S. pneumoniae*, which is associated with conjugative transposon Tn*916* (Croucher et al. 2009). Macrolide resistance can be caused by the presence of *erm* or *mef/mel*, which are found on a variety of gene cassettes that integrate at multiple sites around the genome (D'Aeth et al. 2021). These resistance genes are not present in all *S. pneumoniae* isolates (Croucher et al. 2013a); therefore, annotation accuracy of significant hits will depend on the presence of the gene in chosen references. Even if many linear references are supplied, conflicting alignment and annotations between genomes can make results difficult to interpret. Consequently, the correct interpretation of macrolide resistance PGWAS in this species has proven challenging using previous approaches (Lees et al. 2016). To highlight issues with using linear references for annotation, unitigs associated with either tetracycline or macrolide (represented here by erythromycin) resistance were identified in 616 *S. pneumoniae* genomes with comprehensive minimum inhibitory concentration (MIC) data (Croucher et al. 2015) using pyseer (Lees et al. 2018). A core genome phylogeny was generated using ggCaller for each antibiotic data set and used for pyseer population-structure correction (Supplemental Fig. 9). This phylogeny qualitatively highlighted a correlation between gene presence, AMR phenotype, and population structure, as seen in work by Croucher et al. (2013a). Annotations of significant unitigs were compared between ggCaller and the built-in pyseer annotation function using only Spn23F as a reference.

**Figure 7.**
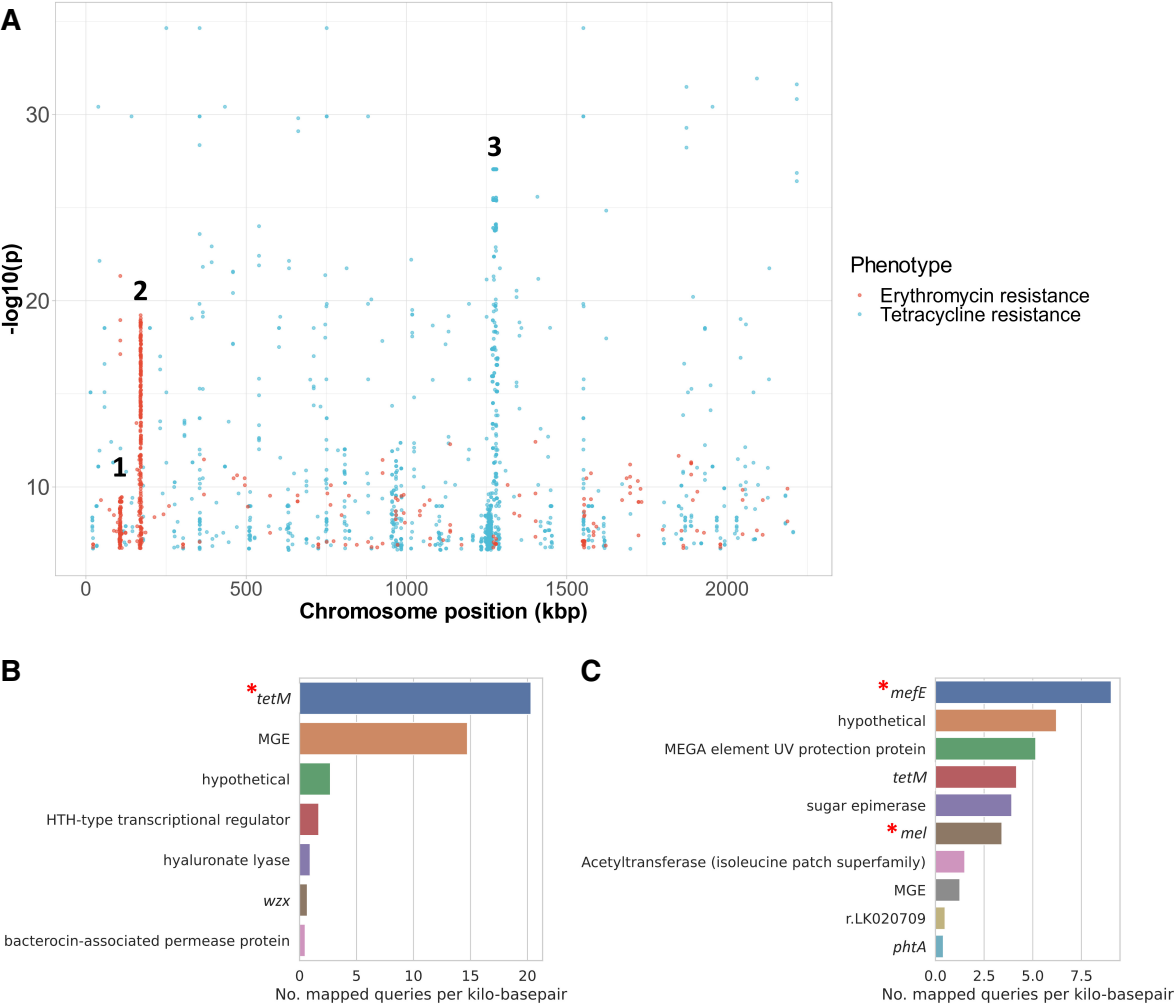
Pangenome-wide association study (PGWAS) of tetracycline and erythromycin resistance. (*A*) Manhattan plot of unitigs mapped to Spn23F. Locus tags and gene names for features in peaks: (1) Spn23F01130 (*capD*), Spn23F01140 (*epsC*); (2) Spn23F01760 (*ruvA*), Spn23F01770 (*tag*), Spn23F01780 (putative protease); and (3) Tn*916*. Gene coverage by significant unitigs associated with tetracycline (*B*) and erythromycin (*C*) resistance identified by pyseer and mapped by ggCaller, with known causative genes marked by red asterisks. Number of mapped queries per kilobase pair was calculated by binning genes matched to queries by ggCaller with the same annotation, and then taking the ratio of the number of queries mapped to the total sequence length of the bin. Core genome phylogenies with resistance and causal gene annotations generated by ggCaller are available in Supplemental Figure 9.

This PGWAS identified a total of 1550 and 726 significant unitig hits for tetracycline and erythromycin resistance, respectively. Mapping these hits to a single reference (Fig. 7A) showed a strong signal at Tn*916* (peak 3) for tetracycline resistance, which contains *tetM*. In contrast, two weaker signals were present at loci associated with erythromycin resistance (peaks 1 and 2). Based on Spn23F annotation, peak 1 aligns to a locus containing the glycosyltransferase, *capD* (locus tag Spn23F01130), whereas peak 2 aligns to a locus containing the DNA-3′-methyladenine glycosylase I, *tag* (locus tag Spn23F01760). Both loci have been identified as insertion sites for Tn*1207.1*-type elements that can harbor *mef/mel* genes (D'Aeth et al. 2021). As Spn23F does not contain *erm* or *mef/mel*, these peaks are false-positive hits resulting from linkage disequilibrium between homologs of *capD* and *tag*, and loci associated with erythromycin resistance. Even if multiple references were supplied that did contain *erm* or *mef/mel*, spurious matches to these loci in Spn23F would make interpretation challenging. In contrast, mapping significant unitigs to DBGs annotated by ggCaller correctly and directly identified the causal genes. Genes annotated as *tetM* had the greatest coverage of significant unitigs associated with tetracycline (Fig. 7B), whereas genes annotated as *mefE* were at the top for erythromycin, with *mel* having the sixth highest coverage (Fig. 7C). No significant unitigs mapped to *erm* genes, which was likely owing to a lack of statistical power as fewer isolates contained these genes compared to *mef/mel* (Supplemental Fig. 9). Therefore, ggCaller provides a useful extension to PGWAS to avoid incorrect or difficult manual functional inference of hits when restricted by arbitrary reference genome choice.

### ggCaller performance scales with population variation

Existing pangenome analysis workflows rely on iterative and usually redundant annotation of genes within independent genomes. In contrast, ggCaller predicts and annotates genes across a population within a DBG and gene graph, respectively. Therefore, ggCaller computational performance is expected to scale with DBG complexity (given by number of nodes and edges), in turn dictated by population variation, rather than linearly with the number of samples. To understand the effect DBG complexity has on computational performance, we benchmarked ggCaller against Prokka + Panaroo using two *S. pneumoniae* and one *Neisseria gonorrhoeae* data sets, representing different levels of pangenome diversity. Two thousand *S. pneumoniae* genomes from the worldwide Global Pneumococcal Sequencing project (Gladstone et al. 2019) and 500 from the statewide Massachusetts data set from Croucher et al. (2015) were used to represent variable levels of diversity within a single pathogen with moderate pangenome diversity, whereas 3000 *N. gonorrhoeae* genomes from Blackwell et al. (2021) were used to represent a global sample of a pathogen with low pangenome diversity (de Korne-Elenbaas et al. 2022). Gene annotations used by ggCaller and Prokka for *S. pneumoniae* (1,231,479 gene annotations) and *N. gonorrhoeae* (32,056 gene annotations) were retrieved from Croucher et al. (2015) and Unemo et al. (2016), respectively. The number of nodes and edges in DBGs for the global *S. pneumoniae* data set were greater than the *S. pneumoniae* Massachusetts and global *N. gonorrhoeae* data sets for equivalent or fewer sample sizes (Supplemental Fig. 10), highlighting the greater level of diversity present in the global *S. pneumoniae* population.

**Figure 8.**
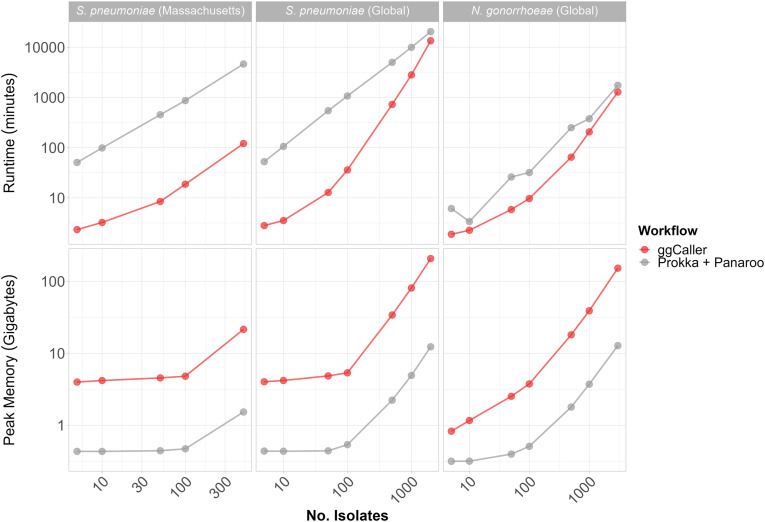
Computational benchmarking of ggCaller against workflow of Prokka + Panaroo. Tools were run using 16 threads, comparing runtime (*top*) and peak memory (*bottom*), with an increasing number of randomly sampled genomes. *Horizontal* panels describe data set *S. pneumoniae* (Massachusetts), data set from Croucher et al. (2015); *S. pneumoniae* (global), data set from Gladstone et al. (2019); and *N. gonorrhoeae* (global), data set from Blackwell et al. (2021). For consistency, the same gene annotation databases were provided to both Prokka and ggCaller for each data set.

ggCaller runtime was reduced compared to Prokka + Panaroo for all data sets (Fig. 8), although the degree of speed-up was data set and sample-size dependent. For example, at 100 genomes, ggCaller was 29.7-fold and 46.4-fold faster than Prokka + Panaroo for the *S. pneumoniae* global and Massachusetts data set, respectively, decreasing to 6.9-fold and 38.3-fold at 500 genomes (Supplemental Fig. 11). In comparison, there was less of a reduction in runtime for the *N. gonorrhoeae* data set, reaching 3.3-fold and 3.9-fold improvements for 100 and 500 genomes, respectively. Nevertheless, ggCaller was faster when run on thousands of genomes, achieving a 1.3-fold and 1.5-fold speed-up for 2000 *S. pneumoniae* and 3000 *N. gonorrhoeae* genomes, respectively. For Prokka + Panaroo, the bulk of processing time was spent during Prokka gene annotation, even with parallelization (Supplemental Fig. 12). This process relies on individual annotation of genes in each genome through BLAST and HMM searches, resulting in repeated computation, rather than sharing annotations across orthologs in ggCaller. Prokka runtime was shown to be dependent on annotation database size (Supplemental Table 4). Therefore, the reduced ggCaller speed-up observed for *N. gonorrhoeae* compared with *S. pneumoniae* is attributable to smaller annotation databases. Smaller annotation databases favor Prokka in terms of runtime as the speed gain from per-COG annotation in ggCaller, rather than per-gene annotation in Prokka, will be reduced. We therefore expect a greater speed-up for ggCaller over Prokka when using large annotation databases for equivalent data sets. However, because of superlinear scaling versus linear scaling between ggCaller and Prokka + Panaroo, respectively, linear gene prediction and annotation will eventually outperform ggCaller. The number of genomes required to reach this point will be data set dependent, as ggCaller runtime is dependent on both the number of nodes and edges within the DBG (Supplemental Fig. 10) and so will vary with population diversity.

In contrast to runtime performance, ggCaller memory use was always higher than Prokka + Panaroo (Fig. 8). This is owing to ggCaller storing the DBG and gene-calls in memory for fast access, leading to increasing memory usage as population size increases. ggCaller memory usage also scaled with population diversity; peak memory reached 206 GB for 2000 *S. pneumoniae* genomes*,* in contrast to 152 GB for 3000 *N. gonorrhoeae* genomes. Furthermore, ggCaller memory usage scaled superlinearly with DBG complexity and number of isolates (Supplemental Fig. 10), indicating that graph complexity, and by extension pangenome diversity, impacts performance. Overall, ggCaller shows reduced runtime versus an existing pangenome analysis workflow, with its performance scaling with population diversity.

To determine how data set size impacts prediction and clustering consistency, within-COG CV and COG size were compared across the different data sets with increasing numbers of genomes. Results highlighted that ggCaller maintains a greater level of consistency in terms of within-COG gene lengths with an increasing number of genomes over other workflows, while still maintaining large clusters (Supplemental Fig. 13). This was most notable with *N. gonorrhoeae*, indicating that ggCaller reduces variability in gene predictions, particularly in lower diversity pathogens. PEPPAN had the lowest CV of all tools; however, the number of genes within COGs was markedly lower than ggCaller and Panaroo for the *S. pneumoniae* and *N. gonorrhoeae* global data sets. Furthermore, ggCaller core genome estimates were the largest of all tools when analyzing the maximum number of genomes in each data set (Supplemental Fig. 14). ggCaller accessory genome estimates were similar to Panaroo, which consistently estimated the smallest pangenome, likely owing to removal of erroneous COGs.

## Discussion

In recent years, there has been increased focus on improving the accuracy, functionality, and sensitivity of bacterial gene annotation, as well as the overall usability of software tools. Prokka (Seemann 2014), DFAST (Tanizawa et al. 2018), and Bakta (Schwengers et al. 2021) were all developed over the past decade as stand-alone tools that combine gene prediction and functional annotation. However, innovation in the underlying algorithms for gene prediction has stalled; all of the above tools rely on Prodigal for gene prediction (Hyatt et al. 2010). Moreover, bacterial genomes are now no longer analyzed in isolation; data sets of hundreds or thousands of sequences are routinely generated and analyzed at once (Land et al. 2015). Existing pangenome analysis tools already use information provided from the simultaneous analysis of many genomes to improve accuracy (Tonkin-Hill et al. 2020; Zhou et al. 2020; Jonkheer et al. 2022). However, the upstream process of bacterial gene prediction and annotation is still conducted on individual genomes. Therefore, there is huge redundancy and potential for inconsistent prediction when annotating the same gene across multiple genomes. These issues lead to longer runtimes and inaccurate clustering, ultimately impacting inferences made on population structure and gene distributions (Tonkin-Hill et al. 2020; Zhou et al. 2020; Dimonaco et al. 2022; Tonkin-Hill et al. 2023).

We developed ggCaller to leverage population-frequency information to improve the accuracy and speed of gene identification, annotation, and pangenome analysis. ggCaller predicts and annotates genes within a pangenome DBG built from thousands of individual genomes. Sequence sharing, encoded as node frequencies by the DBG, enables several innovations in ggCaller over existing tools: (1) Contig breaks can be traversed using identical paths present in other assemblies; (2) ORF start site frequencies are used to consistently predict start codons; (3) ORF scores generated by Balrog temporal convolutional networks (Sommer and Salzberg 2021) are shared across COGs during ORF filtering; (4) genes are functionally annotated within COGs; and (5) an updated version of Panaroo is implemented for iterative gene clustering, paralog identification, removal of erroneous CDSs, and reidentification of genes missed on the first pass.

ggCaller outperformed existing state-of-the-art tools when applied to a diverse set of simulated and real bacterial data sets containing thousands of genomes. Gene predictions were more consistent in terms of start and stop codon identification and within-COG sequence identity, leading to more accurate clustering and gene frequency distributions. ggCaller was also less sensitive to highly fragmented assemblies than existing tools, enabling greater recall of full-length genes. In terms of computational performance, ggCaller had a reduced runtime against a workflow of Prokka and Panaroo by removing redundancy in scoring and annotation.

ggCaller is a useful addition to PGWAS, enabling reference-agnostic functional annotation when used alongside tools such as pyseer (Lees et al. 2018) or DBGWAS (Jaillard et al. 2018). ggCaller has a streamlined workflow for DBG annotation, core-genome phylogeny generation, and significant hit annotation. When applied to *S. pneumoniae* PGWAS of two AMR phenotypes, ggCaller provided a simple, accurate functional interpretation of significant hits. In contrast, using a single linear reference required expert knowledge of the species’ genome biology and relevant literature and highlighted that the functional interpretation of significant hits can be greatly affected by choice of reference sequence. By extension, ggCaller can be used by any study linking sequence to phenotype, such as in pangenome-wide epistasis analysis (Pensar et al. 2019), or in the development of models for phenotype prediction from genomic data (Lees et al. 2020). Such applications can also include the association of structural variants with a phenotype to increase the statistical power of PGWAS, enabled by Panaroo (Tonkin-Hill et al. 2020). Furthermore, the identification of shared structural variants could allow inference of potential horizontal gene transfer events to investigate recombination and transfer of mobile genetic elements within a species, although this is outside the scope of this current work.

A technical limitation of the current version of ggCaller is its memory usage, as the DBG and all gene-calls across the population are stored for fast access. However, both runtime and memory usage varied depending on the choice of data set. Pangenome diversity is a key factor in ggCaller scalability, as including more variation will increase DBG complexity. A less diverse data set (e.g., a single sequence type or clonal complex) will see the greatest improvement in runtime with ggCaller over current state-of-the-art workflows, alongside less extreme memory usage. More diverse data sets (e.g., global collection of sequence types) will still likely see a speed-up using ggCaller, albeit the effect will be reduced. This scaling with graph complexity places ggCaller in a unique position among pangenomic analysis workflows, meaning it is well suited for the analysis of more closely related isolates, such as in regional surveillance. Annotation database size will also determine relative speed-up compared with linear gene-annotation tools, as ggCaller will perform proportionally fewer queries in a larger database. Newer annotations tools such as DFAST and Bakta use faster sequence-querying methods than Prokka (Tanizawa et al. 2018; Schwengers et al. 2021), although they will suffer from the same scaling issue as each genome is annotated independently. Further work will aim to improve scalability of ggCaller, particularly with memory usage, which can be addressed using memory mapping to leverage low-latency storage media.

Additionally, ggCaller cannot yet be run iteratively, requiring the full complement of genomes to be supplied at the start of analysis. This “online” functionality is available in DBG-based software such as Pantools (Sheikhizadeh et al. 2016) and Bifrost (Holley and Melsted 2020), and is a desirable feature for epidemiological tools, as it enables linear scaling as new genomes are added to data sets. Moreover, an alternative function of ggCaller is gene prediction in unassembled data sets, as Bifrost DBGs can be built from reads (Holley and Melsted 2020). However, graphs from read data are complex and contain paths that do not represent real sequences, and so this was not tested here. Finally, ggCaller is limited to identification of bacterial CDSs, meaning annotation of nonbacterial genes and noncoding RNA is not currently supported.

ggCaller is a novel bacterial gene annotation and pangenome analysis tool that outperforms existing state-of-the-art tools in terms of both speed and accuracy, achieved through its use of pangenome DBGs. ggCaller also enables reference-agnostic functional inference, making it an important extension to PGWASs. Graph-based analysis has the potential to become the new convention in bacterial genomics, bringing with it benefits of reduced redundancy, increased consistency, and improved accuracy over linear-genome-based methods. Enabling graph-based annotation and pangenome analysis is an important step in this transition.

## Methods

### Bacterial data sets used for benchmarking

Seven simulated populations of 100 genomes were generated using the infinitely many genes simulation model (Baumdicker et al. 2010), with the *S. pneumoniae* ATCC 700669 serotype 23F (termed “Spn23F,” NCBI GenBank (https://www.ncbi.nlm.nih.gov/genbank/) accession FM211187.1) (Croucher et al. 2009) reference genome as the root. This process is available as a custom script, which was used previously in the validation of Panaroo (simulate_full_pangenome.py). Parameters of each simulation are detailed in Supplemental Table 5. For the contaminated simulation, random 10-kb fragments of the *S. epidermidis* ASM764v1 chromosome (GenBank accession AE015929.1), which is a common contaminant, were inserted into each assembly. For the fragmented simulation, assemblies were sheared based on real contig fragment lengths from assemblies in Croucher et al. (2015). For each simulation, FASTA files containing simulation assemblies and ground-truth CDS annotations were generated. Illumina paired-end reads were then simulated from all assemblies using ART v2.5.8 (Huang et al. 2012) and assembled using SPAdes v3.15.3 (Bankevich et al. 2012).

*S. pneumoniae* genomes (N = 616) were gathered from Croucher et al. (2015). A representative subset of genomes from a data set of *E. coli* (N = 162) was gathered from an analysis in Lees et al. (2019), originally from Kallonen et al. (2017). *M. tuberculosis* genomes (N = 219) were also gathered from Lees et al. (2019), originally from Cohen et al. (2015).

### Linear-genome gene annotation

For linear-genome-based pangenome analysis, genes were called using Prokka v1.14.6 (Seemann 2014) or GeneMarkS-2 v1.24 (Lomsadze et al. 2018). For Prokka, gene annotation used FASTA-format files as the “trusted” CDS set (“‐‐protein”) if available, and tRNA and rRNA calling was turned off (“‐‐notrna,” “‐‐norrna”). For GeneMarkS-2, genes were called using the online tool version (available at http://exon.gatech.edu/genemark/genemarks2.cgi) with default parameters.

### Pangenome analysis

Linear-genome pangenome analyses were conducted using Roary v3.13.0 (Page et al. 2015), Panaroo v1.2.10 (Tonkin-Hill et al. 2020), or PEPPAN v1.0.6 (Zhou et al. 2020) using gene annotations in GFF format provided by Prokka or GeneMarkS-2. All tools were run using default parameters, with the exception of Panaroo, which was run in sensitive, moderate, and strict modes. ggCaller v1.3.4 was run on assemblies in FASTA-format in either sensitive, moderate, or strict modes. For simulated data sets, results were analyzed using a custom script (compare_simulated_gene_pa.Rmd). For real data sets, gene frequency distributions were compared by generating histograms from gene presence/absence matrices in Rtab format from each workflow.

### Contig break analysis

Five manually annotated pneumococcal capsular polysaccharide synthesis operons from Bentley et al. (2006) were downloaded (GenBank accessions CR931662.1, CR931663.1, CR931664.1, CR931665.1, CR931666.1). To fragment the operons, a single contig break was generated randomly in each manually annotated CDS using a custom script (fragment_at_gene.py). Gene predictions from each operon were compared with ground-truth gene sequences using a custom script (gene_recall.py). This script matches the 3′ ends between ground-truth and predicted genes to determine the number of correctly predicted complete sequences, and calculates the total proportion of ground-truth CDSs covered by gene predictions.

### Gene start/stop site comparison

Amino acid sequences for proteins within Pbp1a, Pbp2b, PsrP, and PspA COGs were extracted from ggCaller and Panaroo analyses of 616 *S. pneumoniae* genome sequences from Croucher et al. (2015). Sequences were aligned to reference protein sequences from Spn23F (Croucher et al. 2009) using MAFFT v7.310 (Katoh et al. 2002). A custom script was used to identify soft-clipping at the start and end of alignments compared with Spn23F sequences (gene_end_comparison.py). This script was also used to conduct all-by-all pairwise alignments within each COG to calculate the average AAI, the proportion of matching amino acids over the gapped alignment length (Doolittle 1981; Raghava and Barton 2006).

### Pangenome-wide association studies

Six hundred sixteen *S. pneumoniae* genomes and their associated AMR MIC data were downloaded from Croucher et al. (2015). Genomes for which MIC data were available for tetracycline and erythromycin were extracted and analyzed as separate data sets for each antibiotic. Isolates were labeled as either susceptible or resistant based on MIC cut-offs: 325/616 genomes had tetracycline MIC data, and 36 isolates were labeled as resistant (MIC ≥ 8 µg/mL) (Ousmane et al. 2018); 604/616 had erythromycin MIC data, and 122 were labeled as resistant (MIC ≥ 1 µg/mL) (Zhou et al. 2012). Unitigs were identified in respective data sets using unitig-caller v1.2.1 (Lees et al. 2020). ggCaller was used to generate core-genome neighbor joining trees that were then midpoint-rooted. Unitigs and neighbor-joining trees were used to train mixed effects models with pyseer v1.3.10 (Lees et al. 2018) for the respective data sets. Significant unitigs were identified using thresholds calculated by a built-in pyseer script (count_patterns.py): 2.42 × 10^−07^ and 1.96 × 10^−07^ for tetracycline and erythromycin, respectively. All significant unitigs were mapped via exact alignment to Spn23F using the built-in pyseer annotation function (annotate_hits_pyseer.py) and to ggCaller-annotated DBGs using query mode in exact mapping (“‐‐query-id 1.0”) for the respective data sets.

Mappings to Spn23F were visualized in Phandango (Hadfield et al. 2018). Mappings to the ggCaller graphs were analyzed using a custom script (count_annotations.py), which determines the coverage of gene annotations by significant hits. Genes missing annotations were marked as “hypothetical,” whereas those annotated as transposons, insertion sequences, integrases, or conjugative elements were marked as a mobile genetic element (MGE). To identify the genes with the greatest coverage of significant unitigs, genes with the same annotation were binned together, and the ratio of the total number of mapped queries to the total number of base pairs within each bin was calculated. This statistic is similar to fragments per kilobase pair of transcript used in differential expression analysis (Zhao et al. 2021).

### Computational benchmarking

Genomes in FASTA-format from the the Global Pneumococcal Sequencing project (Gladstone et al. 2019), *S. pneumoniae* Massachusetts data set from Croucher et al. (2015), and *N. gonorrhoeae* from Blackwell et al. (2021) were randomly sampled using a custom script (sample_genome_lists.py). Files were incrementally added to the subsample to increase data set sizes. As Bifrost removes *k-*mers containing ambiguous bases, resulting in disjointed graphs, assemblies were not included in analyses if they contained one or more ambiguous bases. The same subsampled FASTA files were used for the comparison of all workflows. All workflows were run with 16 threads on a server with 768-GB memory and 2 × 20 core Intel Xeon Gold CPUs. The same sets of CDS annotations were provided for both ggCaller and Prokka to ensure consistency in annotation processes; annotations were gathered from Croucher et al. (2015) and Unemo et al. (2016) for *S. pneumoniae* and *N. gonorrhoeae*, respectively.

### Software availability

ggCaller source code is available at GitHub (https://github.com/samhorsfield96/ggCaller) under the open-source MIT license and as a Supplemental Code. All analysis scripts, as well as instructions on how to use them, used in the manuscript are available at GitHub (https://github.com/samhorsfield96/ggCaller_manuscript) and as Supplemental Code. Source code, data, and analysis scripts are also available on Zenodo (https://doi.org/10.5281/zenodo.8054555). ggCaller v1.3.4 was used for all analysis and is available as a release on GitHub (https://github.com/samhorsfield96/ggCaller/releases/tag/v1.3.4). ggCaller documentation is available from readthedocs: https://ggcaller.readthedocs.io/en/latest/

## Supplementary Material

Supplement 1

Supplement 2
