# Supplementary material for: Accurate and fast graph-based pangenome annotation and clustering with ggCaller
Source: Genome Res. 2023 Sep;33(9):1622–37. doi: 10.1101/gr.277733.123 (PMC10620059; doi:10.1101/gr.277733.123)
Supplement: Supplement 2 [file Supplemental_Material.pdf]

Supplemental Materials for

Accurate and fast graph-based pangenome annotation and clustering with ggCaller

Samuel T. Horsfield, Gerry Tonkin-Hill, Nicholas J. Croucher, John A. Lees

Correspondence to: s.horsfield19@imperial.ac.uk

Contents

Supplementary Methods 2

Supplementary Figures 7

Supplementary Tables 19

Supplementary References 24

Supplementary Methods

Graph indexing

The de Bruijn graph (DBG) used by ggCaller is generated by Bifrost (Holley & Melsted, 2020). Bifrost was chosen as it is highly scalable to hundreds of thousands of bacterial genomes and has an intuitive C++ API. To build a DBG, Bifrost first generates an index of all *k*-mers in the population using a blocked Bloom filter. $k = 31$ has been shown to provide a good balance of efficiency and sensitivity of detection of shared/divergent sequences in bacterial genomes (Holley & Melsted, 2020), and so is used as the default in ggCaller. Starting with a single *k*-mer, Bifrost queries the presence of its suffix (length= $k - 1$) appended with each of A, C, G and T within the blocked Bloom filter (**Figure 1**, **step 1**). Connections of length $k - 1$ between *k*-mer suffixes and prefixes are treated as edges. Each *k*-mer is assigned colours, which describe in which genomes they are present in. Unitigs, also known as nodes, are generated by merging unbranching *k*-mer paths into a single DNA sequence. Branching paths, described by edges, represent variation within a population. A genome can be regenerated from a DBG by traversing a path made up of nodes assigned with a specific colour.

Once the DBG is built, ggCaller iterates over the DNA sequence in each node and identifies stop codons in each reading frame, storing this information in a six-bit bitset, where each bit represents a reading frame (three forward and three reverse) (**Figure 1**, **step 2**). A bit is set to ‘1’ if at least one stop codon is present in that reading frame, and ‘0’ otherwise. ggCaller stores the node colours in a separate bitset; as the colours of the constituent *k*-mers within a node may not all be identical due to contig breaks (Schulz *et al*., 2022), ggCaller takes the intersection of the colours of the start and end *k*-mers for each node to calculate node frequency. ggCaller additionally determines the frequency of stop codons for each reading frame within the graph by counting the number of set bits. For accurate start site identification, ggCaller also identifies the first base of all start codons, and $k - 1$ downstream bases (k chosen as this is the shortest possible node length). This sequence is then translated into its respective amino acid sequence and further split into l-mers ($l = m / 3$, where m = Bifrost minimiser size, chosen to ensure sufficient coverage of each start site). The coverage of each l-mer is calculated within the population and stored in a robin-hood unordered map to be used using graph traversal. Using amino-acid l-mers reduces the impact of small mutations on the calculation of start site frequency, which would otherwise make start sites of non-identical orthologues appear less frequent.

As Bifrost does not consider sequence contiguity from input genomes when inferring edges, traversal of a Bifrost DBG can result in generation of sequences that are not present in the original genomes (Břinda *et al*., 2021). To prevent this, candidate paths are queried against input genomes to ensure they are present as contiguous sequence in the input. An FM-index is built from each genome in node space (using node identifiers rather than DNA sequence) using kseq (https://github.com/lh3/seqtk) and SDSL v3 (Gog *et al*., 2014), to which paths can be compared during traversal. Building an FM-index using node identifiers rather than DNA sequence greatly reduces the size of the index, reducing the memory and time required to load and search the index.

Stop codon search using iterative graph traversal

ggCaller uses an iterative depth first search (DFS) algorithm to pair stop-codons in the same reading frame (**Figure 1**, **step 3a**). To reduce the search space, ggCaller traverses the DBG colour-by-colour (i.e. sample-by-sample). Starting at a source node containing at least one stop codon, ggCaller iteratively visits all neighbour nodes with the same colour, with each neighbour added to the top of a stack. Each iteration of the algorithm removes the top node from the stack and visits its neighbours with the same colour as the source node. As each neighbour is visited, the complete path in node space is queried against an FM-index of the given colour to ensure the path is also contiguous in the underlying genome (**Figure 1**, **step 3b**). This process is repeated until all stop codons in the source node have been paired with a downstream stop codon in the same reading frame. This is now designated as a complete 'stop-to-stop' path. This process is repeated for each node containing at least one stop codon, and for each colour in the DBG.

ORF identification within stop-to-stop paths

For each complete 'stop-to-stop' path, ggCaller identifies all possible start codons in the path and pairs them with their respective downstream stop codons to generate a list of candidate ORFs (**Figure 1**, **step 4**). Starting at the start site for the longest possible ORF, start positions for consecutively shorter ORFs are compared to the current 'high-scorer', with the score dictated by: **i**) the translation initiation site score given by Balrog, **ii**) the median population coverage for all amino-acid l-mers in the start site, and **iii**) the number of times the start site has been chosen as the high-scorer in orthologous ORFs. For a start position of a shorter ORF to be chosen as a high-scorer, it must 'win' in 2/3 of the above criteria against the previous high-scorer.

To avoid checking start positions of short ORFs that are likely to be incorrect, their start sites are penalised using a probabilistic model. As genes are characterised by long stretches of DNA without two stop codons in the same reading frame, the average frequency of stop codons within genes will be lower than the population average. Therefore, short ORFs can be penalised based on the probability of observing the longer of two ORFs if stop codons occurred at a constant rate (**Equation 1**). This is given by the population stop-codon frequency (p) and the difference in length between the long (l_{long}) and short (l_{short}) ORF. This gives the probability of the longer ORF occurring by chance if stop codons occurred randomly at the population average frequency (q). q is only calculated between the current high-scorer and next start site in the iteration. If q falls below a threshold, meaning the probability of observing the current high scorer is unlikely by chance, all subsequent shorter ORFs are ignored. A default of $q < 0.2$ (i.e. <20% probability of longer ORF occurring by chance) was chosen, as this provides a good trade-off between sensitivity and precision.

Equation 1:

$$q = (1 - p)^{l_{long} - l_{short}}$$

ORF clustering and gene scoring

At this stage, all ORFs have been identified across all colours. However, many of these sequences are similar, so to remove redundancy from downstream scoring and pangenome steps, ggCaller first clusters ORFs using an algorithm with a similar approach to Linclust (Steinegger & Söding, 2018), but adapted for DBG sequences (**Figure 1**, **step 5**). Firstly, ggCaller identifies all sets of ORFs which share at least one node as candidate clusters, placing them in a 'node group', and identifies the largest ORF for each node group as a centre sequence. ggCaller then translates every ORF and aligns them with the centre sequence of the node group(s) it belongs to using Edlib (Šošić & Šikić, 2017). If the comparison surpasses a given identity and length cut-off, the ORF is clustered with the centre sequence. A default of 98% for identity and length cut-off was chosen, as these are the initial clustering parameters used in Panaroo for identifying orthologues. For ORFs that do not cluster with a centre sequence, the process is repeated until each ORF is either paired with a centre sequence, or is a centre sequence itself.

Centre sequences are then scored based on how likely they are to be a real coding sequence by using Balrog (**Figure 1**, **step 6**). Balrog uses a temporal convolutional network which generates an average score per amino-acid residue, which is incorporated with start codon sequence, the length and TIS score to generate an individual score for each ORF in the cluster. ORFs with a score below a pre-defined cut-off (default = 100, as used in Balrog) are removed from the set. Those surpassing the score are regarded as candidate protein coding sequences (CDSs).

CDS filtering

In the final prediction step, overlapping CDSs must be filtered to pick the most likely final gene calls. This is achieved by finding the highest-scoring tiling path through regions of the DBG with overlapping CDSs. Overlaps between CDSs are first calculated for each colour using a sparse matrix generated using Eigen v3 (Guennebaud, Jacob *et al*., 2010). Each row in the sparse matrix is an CDS ID and each column is a node in the DBG, with '1' in each cell where the node is contained within the ORF, and '0' otherwise. This matrix is multiplied by the transpose of itself, resulting in a sparse matrix with CDS IDs on both rows and columns, and non-zero values off the diagonal indicating ORFs that share at least one node and therefore have a potential overlap. ggCaller then iterates over these potential overlaps, and identifies whether each pair of CDSs genuinely overlap, or whether they simply share sequence, such as a repeated motif. Overlaps are calculated using respective DBG coordinates of each CDS. If two CDSs do overlap, then the number of overlapping base-pairs is calculated, as well as the direction of the overlap.

For each set of overlaps, ggCaller then generates a subgraph of these CDSs as a directed graph using the boost graph library (Siek *et al*., 2002), where each node is an CDS, and each edge is an overlap (**Figure 1**, **step 7**). Each node is labelled with the respective score for that CDS, and each edge with the overlap penalty calculated from the size of the overlap and the penalty scores provided by Balrog. The graph is then separated into connected components, and each component is traversed using the Bellman-Ford algorithm (Bellman, 1958; Ford & Fulkerson, 1962) to determine the highest scoring path. The Bellman-Ford algorithm requires two small modifications to the graph: all scores are multiplied by -1, and all cycles in the graph are pruned via removal of back edges (Siek *et al*., 2002). A transitive closure is then generated, which connects all upstream nodes to all downstream nodes that have a viable path between them. This step enables skipping of CDSs mid-way within a path if a higher scoring path is present. CDSs present in the highest-scoring path are returned as 'true' gene-calls.

Pangenome analysis using DBG-based Panaroo

Panaroo builds gene graphs and applies filtering criteria to prune false positive gene calls, and gene refinding to mitigate false negatives. We integrated these steps into ggCaller for the same advantages, and to simultaneously and efficiently produce an annotated pangenome matrix. The original version of Panaroo (Tonkin-Hill *et al*., 2020) operates on linear genome annotations; a gene graph is then generated by CD-hit (Li & Godzik, 2006) iteratively clustering genes, and adjacency information between genes drawn from input assemblies. As it would be redundant to regenerate this information from the above annotations, we implemented a modified version that works directly with the ggCaller annotated graph (**Figure 1**, **step 8**). Genes which do not have existing neighbours from the overlap step are connected to a neighbour via a DFS of the DBG for each colour. All connected genes are used to generate a gene graph, where genes are added to the same nodes if they belong to the same clusters calculated previously. We implemented the same three stringency settings as Panaroo for removing spurious COGs. Spurious COGs are identified as those at the end of contigs (i.e. have 0/1 edges), or are in a separate component to the majority of COGs. Each stringency mode removes spurious COGs below a certain population frequency (strict: <5%, moderate: <1%, sensitive: none removed).

We also included several alterations to the original Panaroo algorithm. After the initial collapse of gene families, each gene cluster is functionally annotated (i.e. predicted gene name and function through similarity search) based on alignment of each cluster's centre sequence to a pre-defined annotation database using DIAMOND (Buchfink *et al*., 2014) and HMMER3 (Eddy, 2009). This annotation is then shared across all members of the cluster. This cluster-by-cluster annotation approach scales more efficiently than existing linear genome annotation tools when analysing many genomes at once. We provide two default annotation databases; UniProt (Bateman *et al*., 2021) (queried 'Bacteria' and 'Virus', only reviewed sequences included) for DIAMOND and HMM profiles from Prokka (Seemann, 2014) for HMMER3. Custom databases can also be provided (amino acid FASTA for DIAMOND, HMM-profiles generated by 'hmmbuild' for HMMER3). Gene-refinding, which is implemented in Panaroo to search for missing gene-calls within linear genomes, has been altered to work within the Bifrost DBG. We also simplified within-cluster alignment, replacing the choice of aligners with only MAFFT. We implemented two options for running MAFFT: **i**) default mode, where all sequences are aligned together, and **ii**) reference mode, where centre sequences are aligned together, and then the remainder of sequences are aligned in reference-guided mode. The former is faster when only a few sequences are present in each cluster, whereas the latter can be used to speed-up alignment of large clusters.

Querying of sequences within an annotated DBG

ggCaller supports querying new sequences against an annotated DBG, which is useful during functional inference of significant hits in pangenome wide association studies. Multiple query sequences can be supplied, which are broken into *k*-mers and queried against the graph using the Bifrost 'query' function. This identifies all node sequences that are found in the query sequence. As all CDSs identified by ggCaller are indexed by node, any that share at least one node with the query sequences are returned. Returned CDSs are output in FASTA format, detailing the query or queries with which they overlap, and the genome(s) in which they are found.

Output files and plots

By default, ggCaller generates FASTA files of all CDSs in nucleotide and amino-acid format. ggCaller also generates the same files as Panaroo (except for gene_data.csv, a summary of CDSs and their annotation, all of which is included in ggCaller FASTA files), including a gene-presence absence matrix, annotated graph in GML format, and per-cluster alignments in FASTA format. If annotation is specified, ggCaller can also generate GFF files for assemblies included in the DBG. We also implemented pairSNP-cpp (https://github.com/gtonkinhill/pairsnp-cpp) and Rapidnj (Simonsen *et al*., 2008) to rapidly generate SNP distance matrices, and neighbour-joining Newick trees of all input genomes. ggCaller also generates within-cluster alignments and concatenated core-genome alignments for phylogenetic analysis. Additionally, ggCaller uses SNP-sites (Page *et al*., 2016) to identify single nucleotide polymorphisms in alignments in VCF format. ggCaller also generates summary graphs, including a rarefaction curve for estimating pangenome 'openness' (Tettelin *et al*., 2005), and gene frequency distributions (Baumdicker *et al*., 2010), either in terms of cluster size or population proportion.

Computational optimizations

ggCaller is written in C++ and Python, and is parallelizable through use of OpenMP (Dagum & Menon, 1998) and Python's multiprocessing module. ggCaller relies on robin-hood hash maps for in-memory storage of ORF graph coordinates, which are more time and memory efficient than standard C++ unordered maps (https://github.com/martinus/robin-hood-hashing). ggCaller reimplements the Balrog model of translation initiation site and gene structure (Sommer & Salzberg, 2021). The temporal convolutional network structure and weights are rewritten using torch to enable direct C++ integration (Collobert *et al*., 2002). These models were initially trained using pytorch and compatible only with a Python API (Paszke *et al*., 2019), with torch reimplementation improving model querying speed and enabling parallelisation using shared memory. ORF scores generated by Balrog are stored in a lock-free dictionary which can be accessed by independent threads to avoid rescoring the same sequence. ggCaller builds an FM-index using node identifiers instead of DNA sequence to identify incorrect paths generated during DBG traversal. This significantly reduces time and memory requirements during FM-index querying.

Supplementary Figures

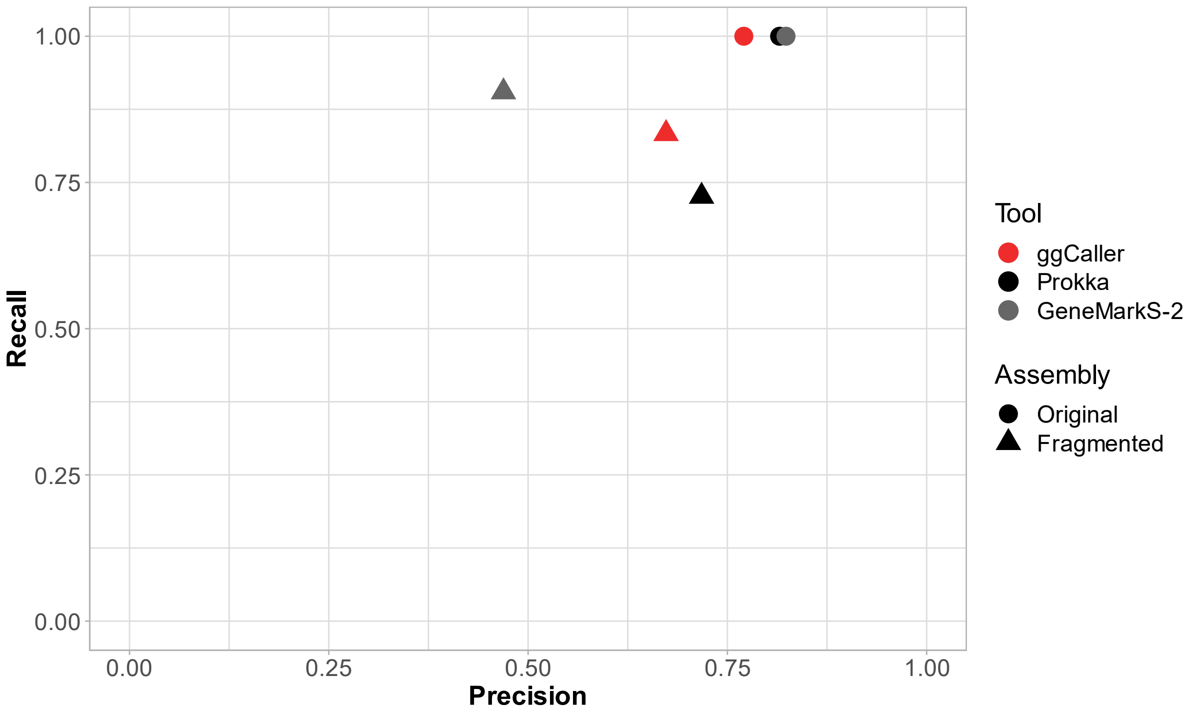

Supplementary Figure 1: 3' accuracy of gene prediction comparison for pneumococcal capsular biosynthetic operons with and without fragmentation. The scatterplot compares precision and recall for gene predictions, for which the 3' end matches that of a ground-truth sequence. A 5' match between the prediction and ground-truth is allowed but not required.

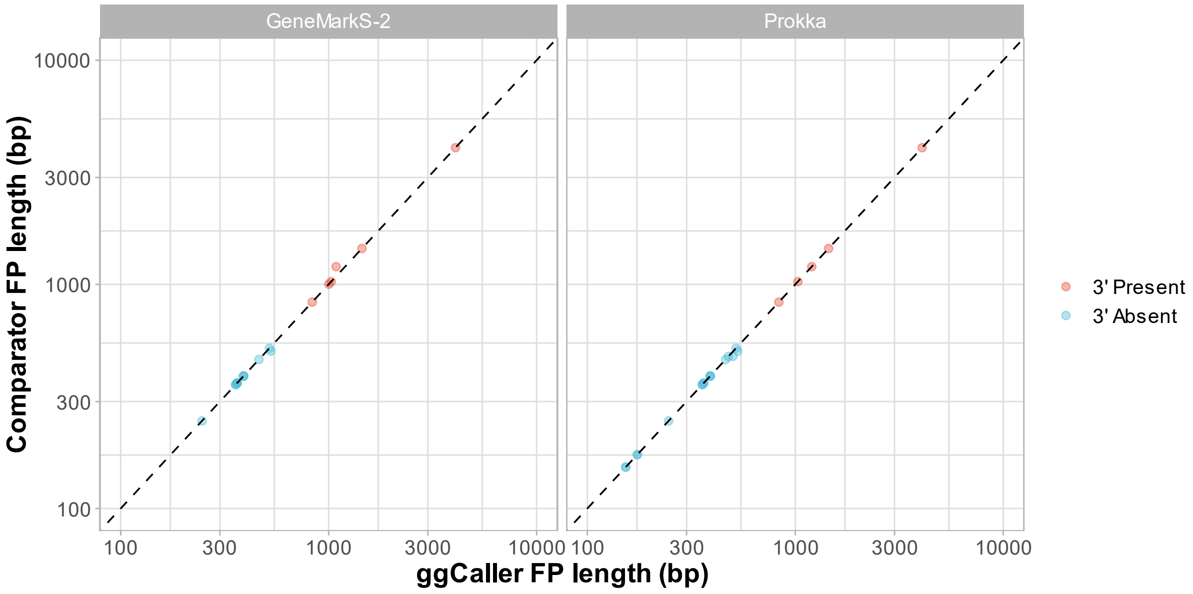

Supplementary Figure 2: Length comparison of shared False positives (FPs) between ggCaller and comparator tools when applied to pneumococcal capsular biosynthetic operons without fragmentation. Horizontal panels describe the comparator gene-prediction tools which share FPs with ggCaller. Colours describe whether the FP matches the 3' end of a ground truth sequence but mismatches at the 5' end (3' Present) or if the FP is a spurious prediction (3' Absent). Dashed line is the line of identity.

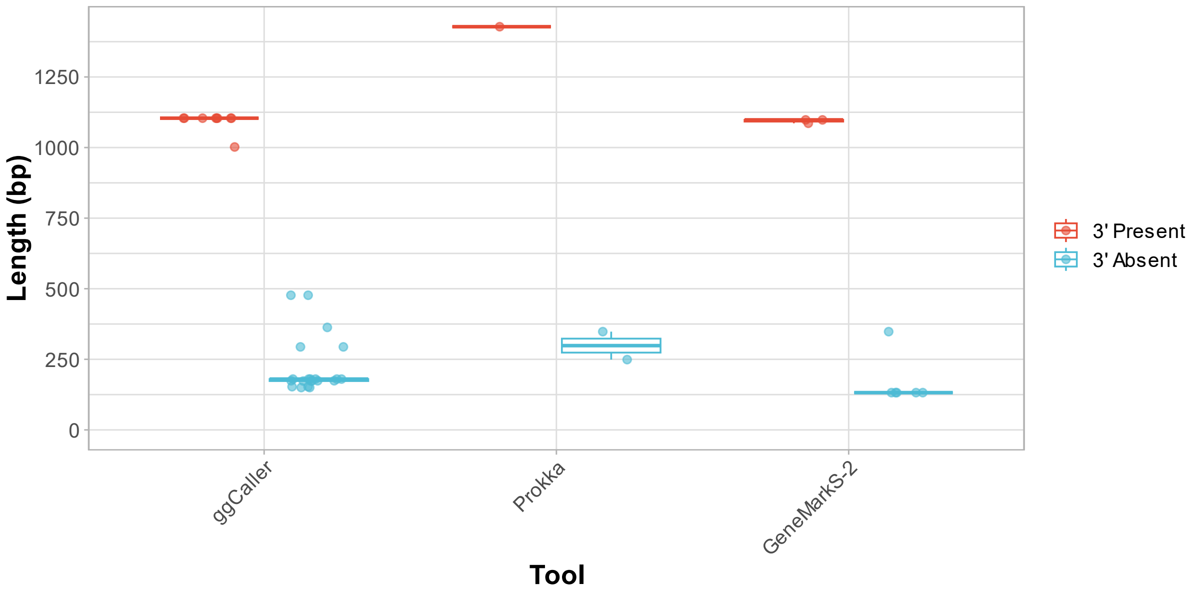

Supplementary Figure 3: Length comparison of False positives (FPs) exclusive to each tool between ggCaller and comparator gene-prediction tools when applied to pneumococcal capsular biosynthetic operons without fragmentation. Colours describe whether the FP matches the 3' end of a ground truth sequence but mismatches at the 5' end (3' Present) or if the FP is a spurious prediction (3' Absent).

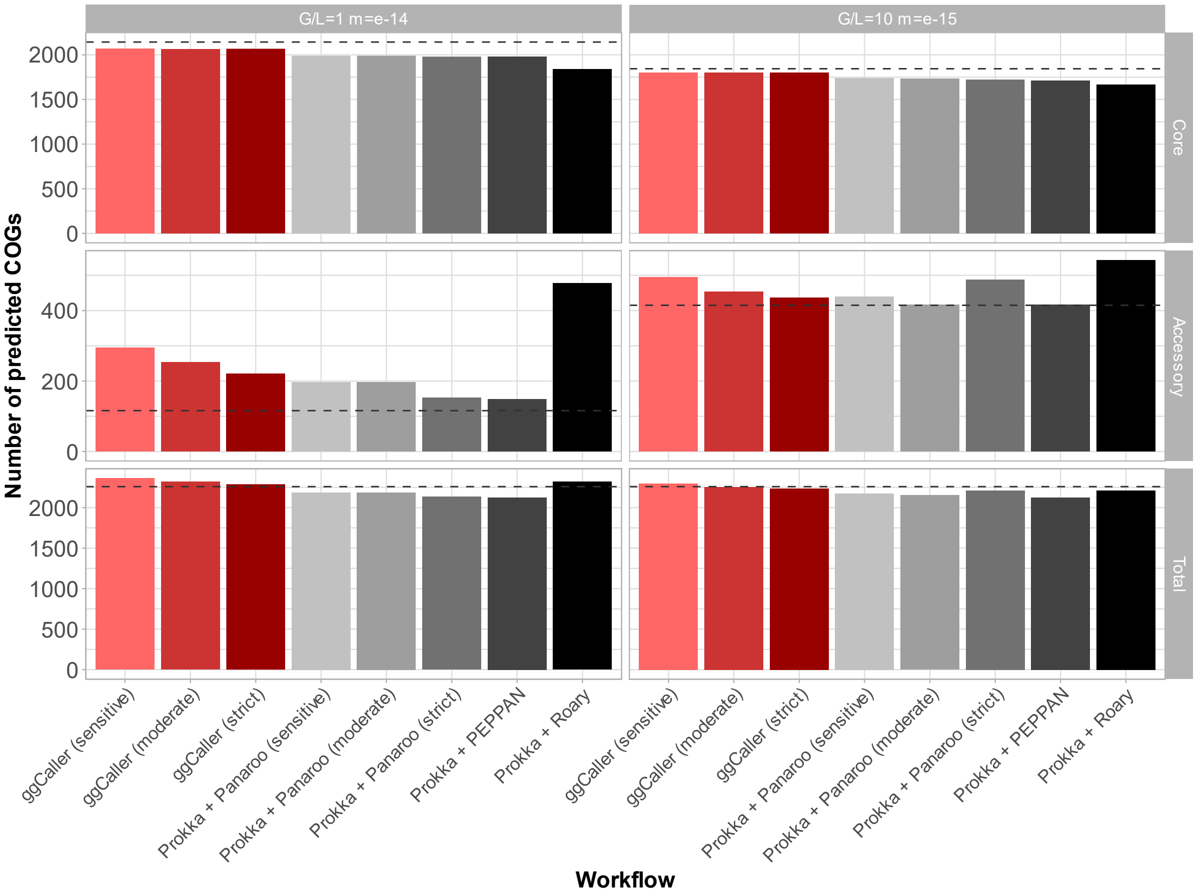

Supplementary Figure 4: Comparison of estimated core, accessory and total pangenome sizes across simulated populations for simulations not included in Figure 3. Panels describe simulations with simple (**A**) and complex (**B**) sources of error. Bars indicate the predicted number of COGs for each workflow. Ground truth values are represented by the grey dotted line in each panel. Horizontal panels describe simulation parameters, vertical panels describe COG frequency; core (99% ≤ x ≤ 100%), accessory (0 ≤ x < 99%) and total (0 ≤ x ≤ 100%).

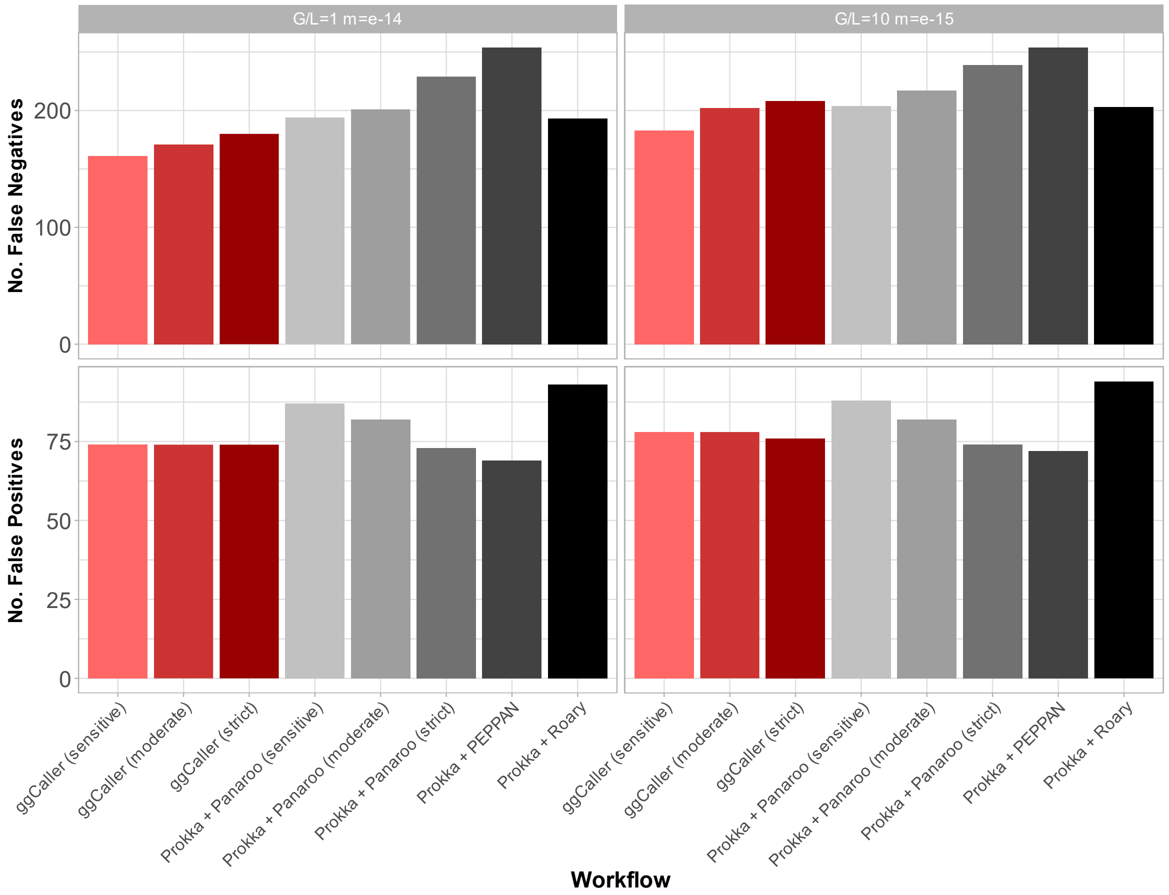

Supplementary Figure 5: Comparison of COG annotation accuracy across simulated populations for simulations not included in Figure 4. False negatives (top) are COGs that were present in the ground-truth set but not called by a workflow. False positives (bottom) are COGs that were called by a workflow but were not present in the ground-truth set.

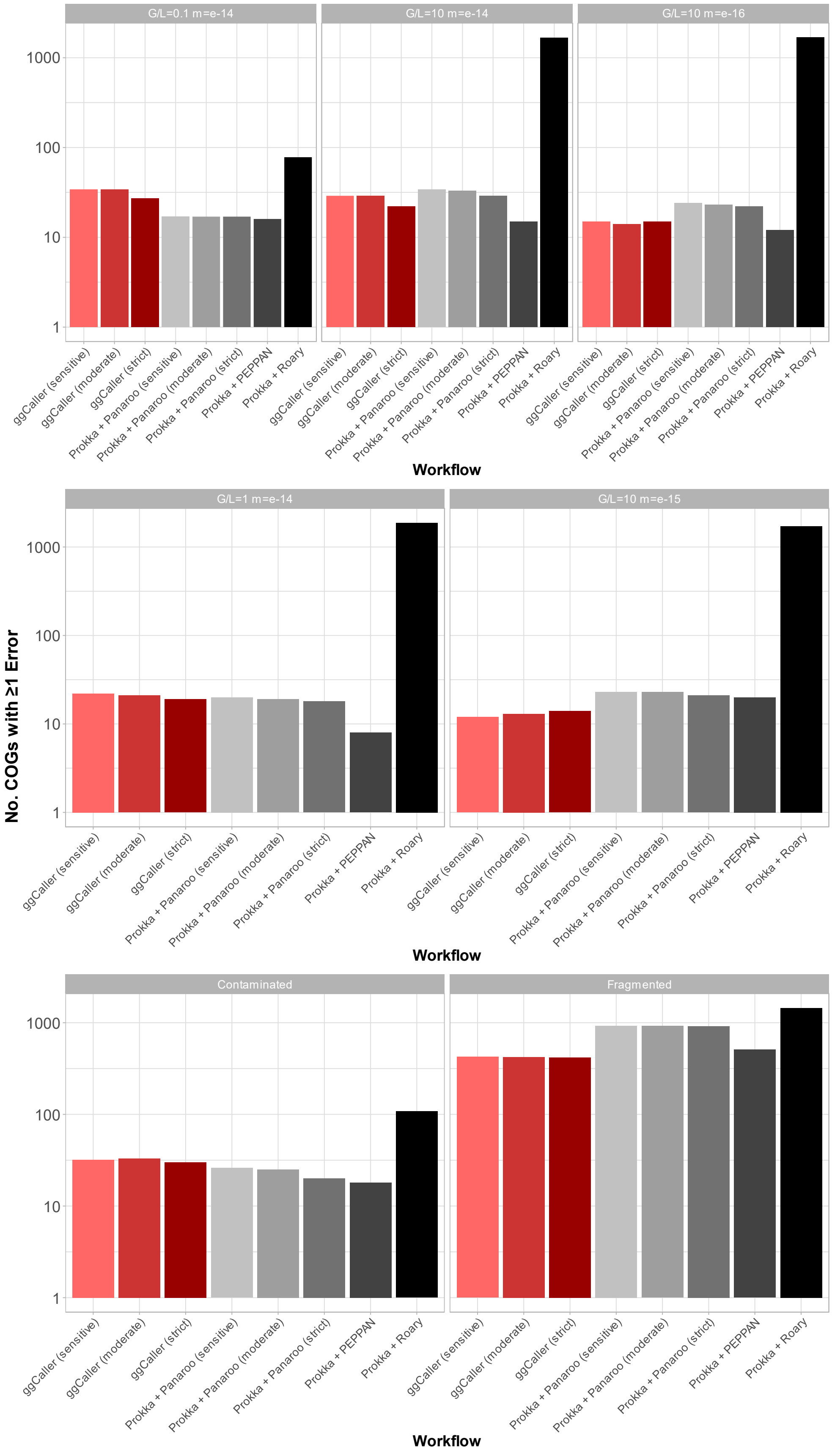

Supplementary Figure 6: Comparison of number of COGs that contain at least one prediction error. For a COG to have a prediction error, it must contain an incorrectly predicted gene in one of the individual genomes. This can either be a gene called by a workflow which is missing in the ground truth (false positive), or a gene that is missed by a workflow present in the ground truth (false negative).

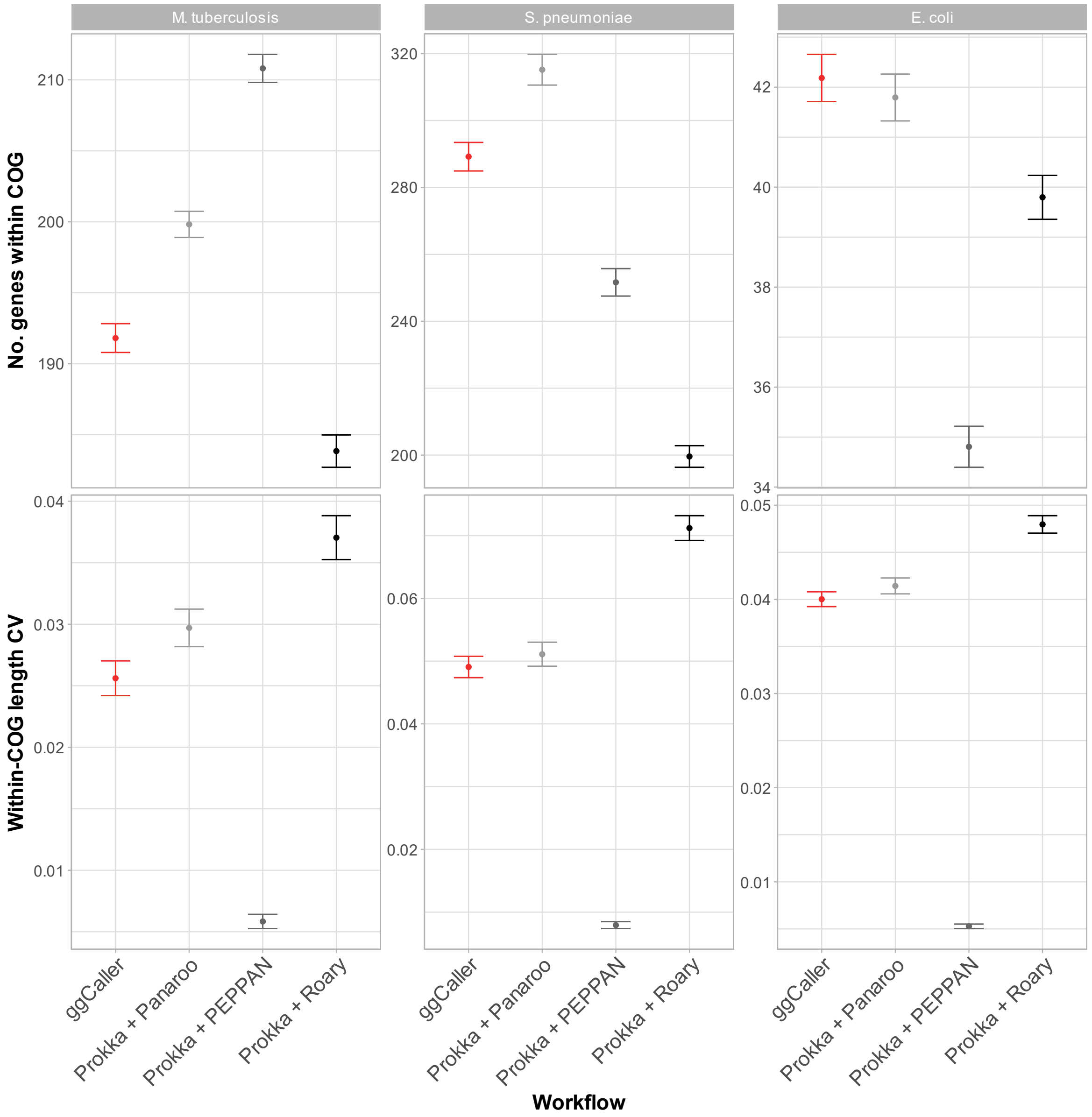

Supplementary Figure 7: COG prediction consistency across pangenome analysis workflows for real bacterial genome datasets. Plots show number of sequences with a COG (**top**) and within-COG Coefficient of Variation (CV) of CDS lengths (**bottom**). Refound genes and sequences annotated as 'pseudogenes' were removed from ggCaller and PEPPAN respectively. Points and bars highlight average and standard error respectively Horizontal panels describe species datasets: *M. tuberculosis* from Cohen *et al*., (2015), *S. pneumoniae from* Croucher *et al*., (2015), *E. coli* from Kallonen *et al*., (2017).

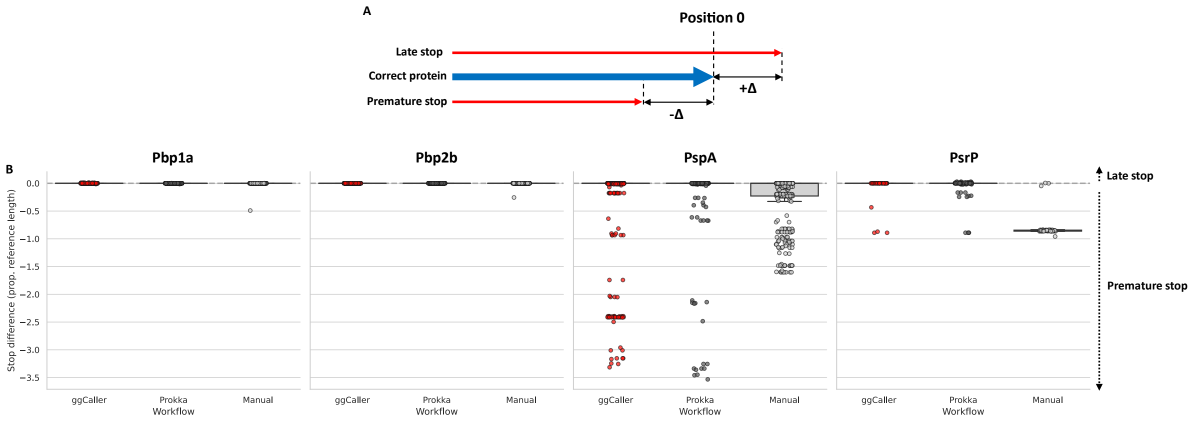

Supplementary Figure 8: Comparison of within-COG stop site soft-clipping across ggCaller, Prokka and the original S. pnuemoniae Massachusetts dataset. (**A**) Description of stop site soft clipping. (**B**) Boxplot comparisons of stop site soft-clipping protein sequences of Pbp1a, Pbp2a, PspA and PsrP based on alignment with the manually annotated reference in Spn23F.

A

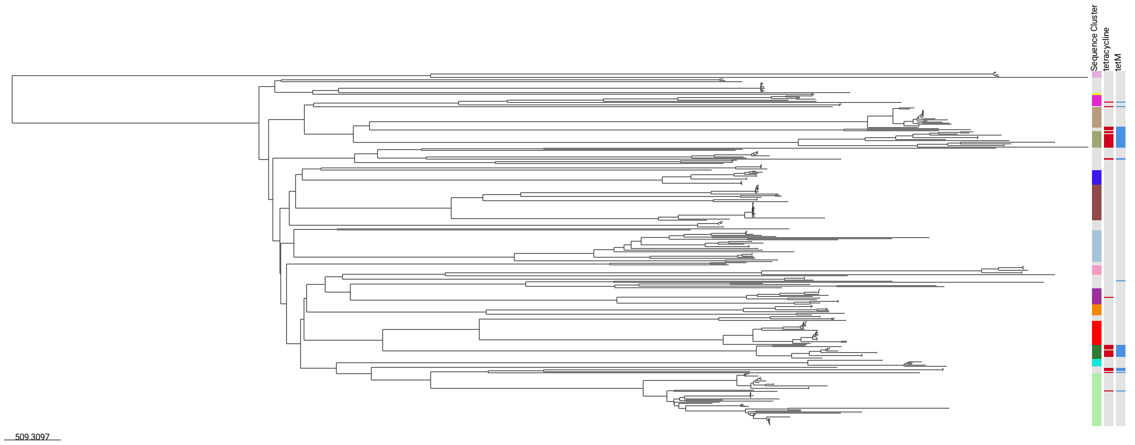

B

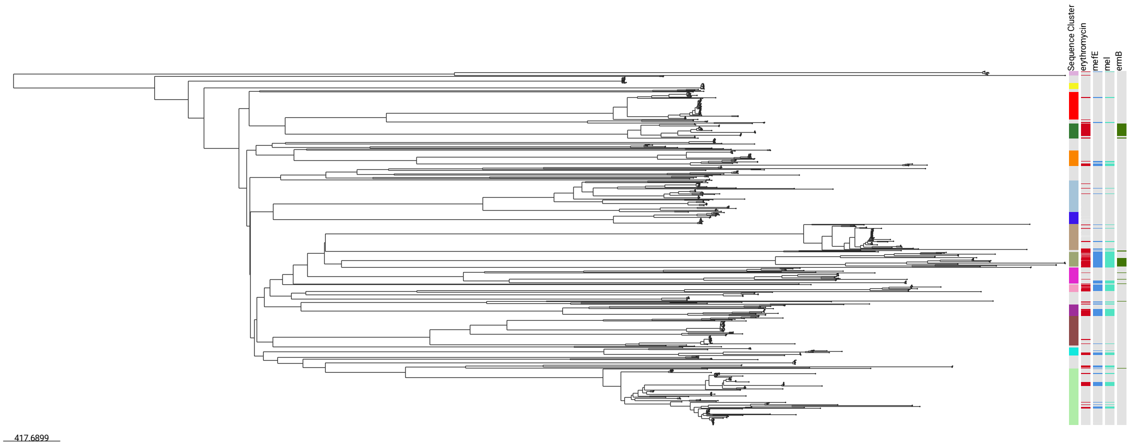

Supplementary Figure 9: Core genome neighbour-joining tree generated by ggCaller annotated with resistance phenotype and AMR gene presence in S. pneumoniae. Trees were built from (**A**) 325 isolates with tetracycline MIC data and (**B**) 604 isolates with erythromycin data (Croucher *et al.*, 2015). Trees were displayed using Microreact (Argimón *et al.*, 2016). Key for core SNP-distance to branch length shown in bottom left corner of each panel. Blocks to right of trees describe sequence clusters assigned in Croucher *et al.*, drug resistance phenotype and AMR gene presence identified by ggCaller.

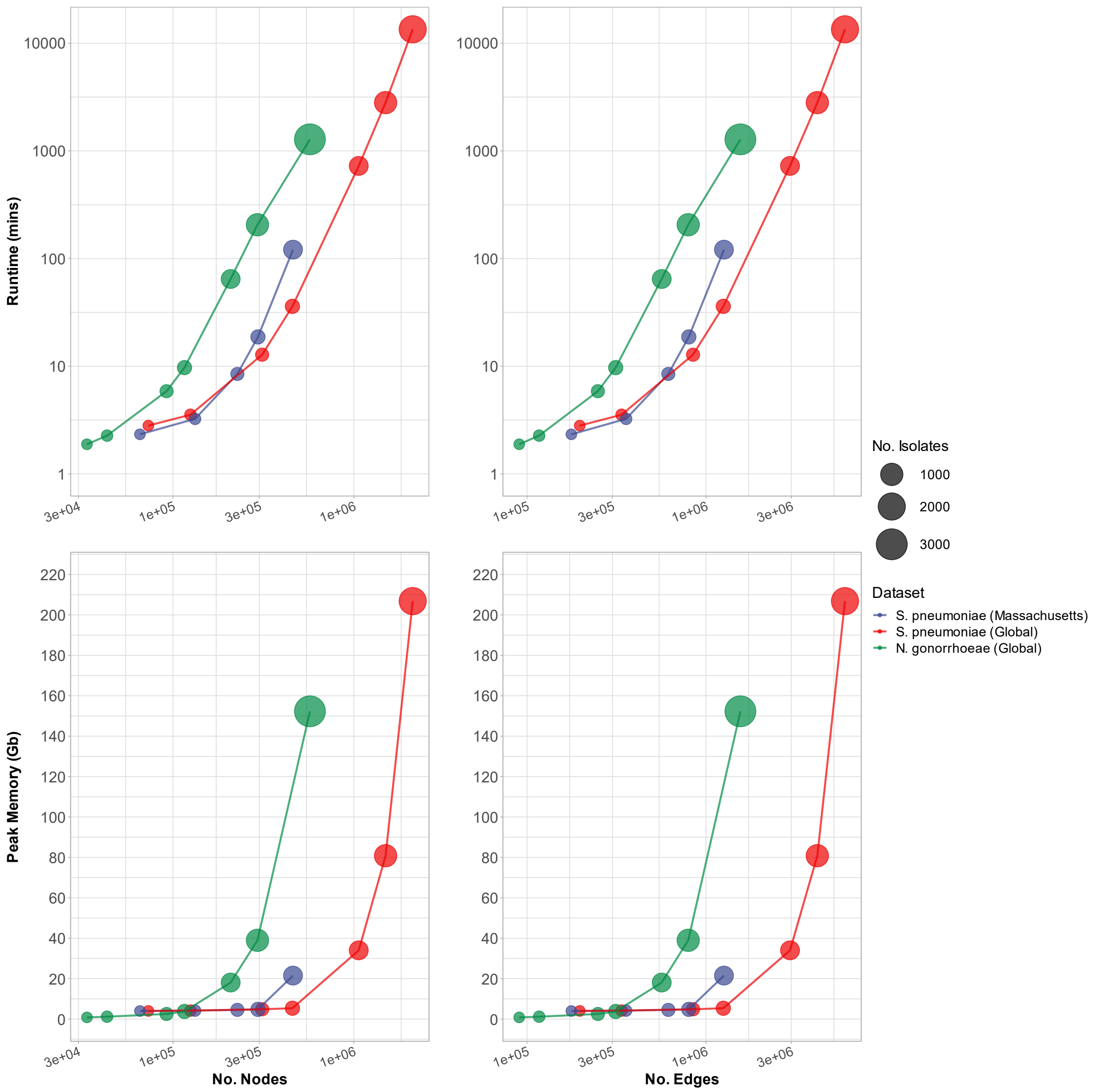

Supplementary Figure 10: Comparison of de Bruijn Graph complexity and ggCaller computational performance. Nodes refer to unitigs within a de Bruijn Graph, edges refer to connections between nodes. Point labels indicate the number of isolate genomes included in analysis. All analyses were run with 16 threads. Colours describe dataset: *S. pneumoniae* (Massachusetts), dataset from Croucher *et al*., (2015); *S. pneumoniae* (Global), dataset from Gladstone *et al*., (2019); *N. gonorrhoeae* (Global), dataset from Blackwell *et al*., (2021).

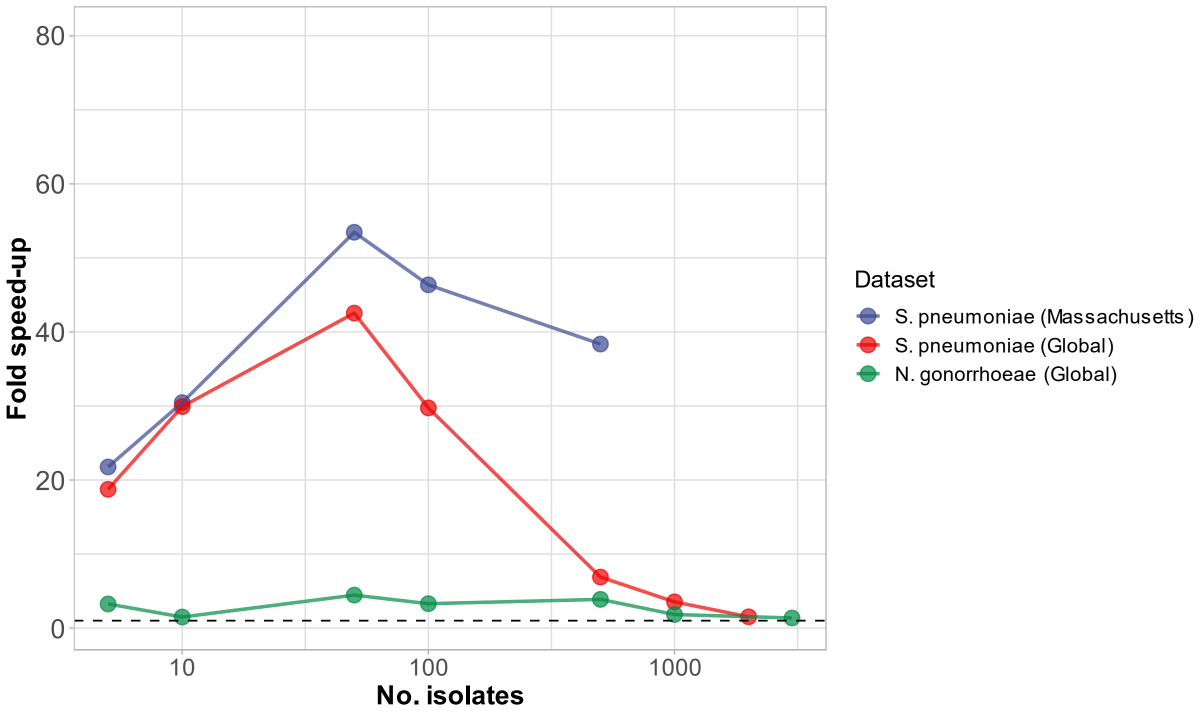

Supplementary Figure 11: Fold speed-up of ggCaller over Prokka + Panaroo for increasing dataset size. Fold speed-up was calculated by dividing the runtime of Prokka + Panaroo by that of ggCaller for runs with identical datasets. Both workflows were run with 16 threads. Colours describe dataset: *S. pneumoniae* (Massachusetts), dataset from Croucher *et al*., (2015); *S. pneumoniae* (Global), dataset from Gladstone *et al*., (2019); *N. gonorrhoeae* (Global), dataset from Blackwell *et al*., (2021).

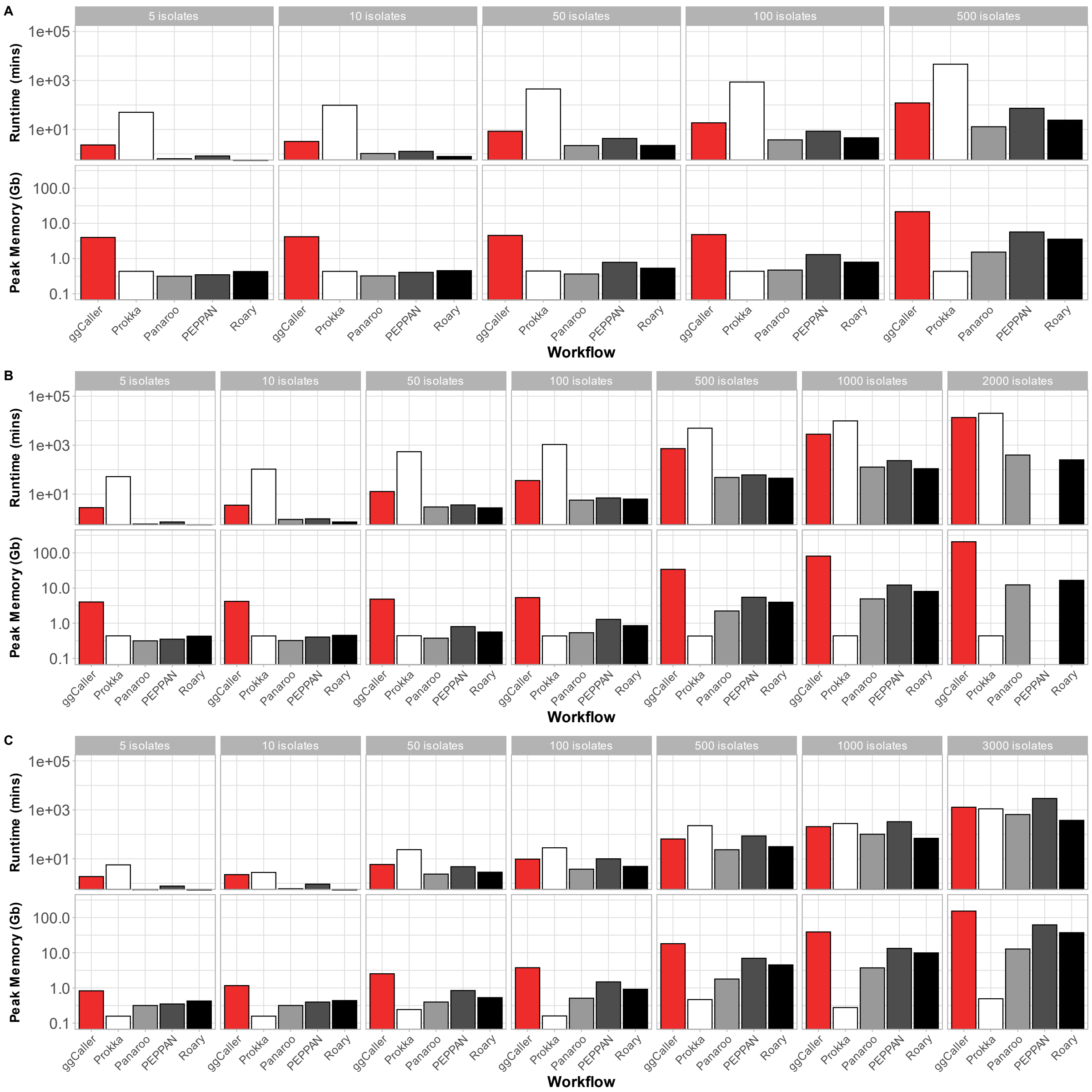

Supplementary Figure 12: Computational performance comparison between ggCaller and pangenome analysis workflows. Workflows were run across three datasets: (**A**) *S. pneumoniae* (Massachusetts), dataset from Croucher *et al*., (2015); (**B**) *S. pneumoniae* (Global), dataset from Gladstone *et al*., (2019); (**C**) *N. gonorrhoeae* (Global), dataset from Blackwell *et al*., (2021). Workflows were split into gene prediction and annotation (Prokka) and pangenome analysis (Panaroo, PEPPAN and Roary), with the exception of ggCaller, which includes all of these processes. PEPPAN did not finish with 2000 Global *S. pneumoniae* genomes. All tools were run using 16 threads.

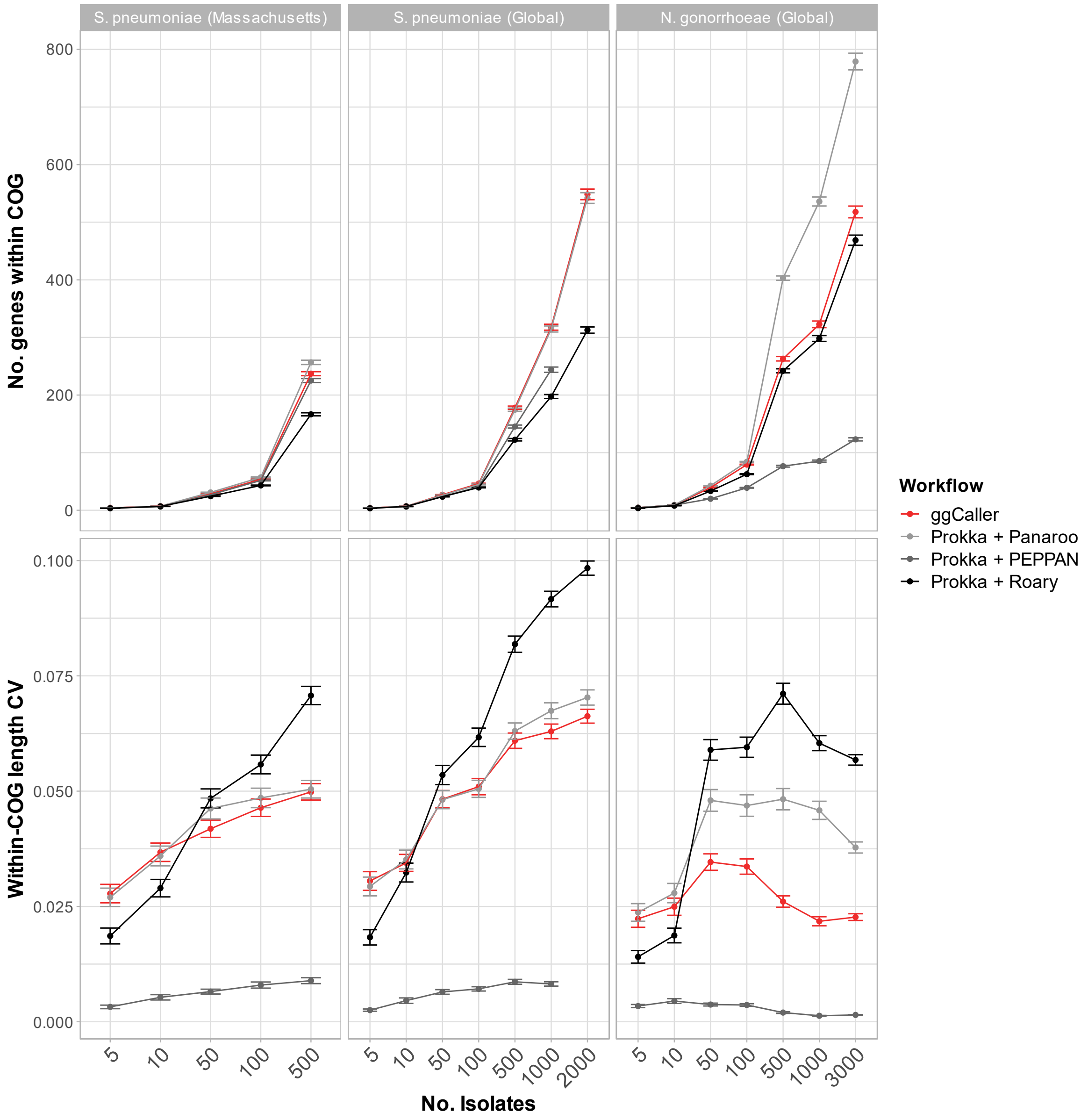

Supplementary Figure 13: Gene prediction consistency across pangenome analysis tools on three pathogen datasets. Workflows were compared on the number of gene sequences within each COG (**top**) and within-COG sequence length coefficient of variation (CV) (**bottom**). Points and bars describe the mean and standard error of each distribution respectively. Horizontal panels dataset: *S. pneumoniae* (Massachusetts), dataset from Croucher *et al*., (2015); *S. pneumoniae* (Global), dataset from Gladstone *et al*., (2019); *N. gonorrhoeae* (Global), dataset from Blackwell *et al*., (2021).

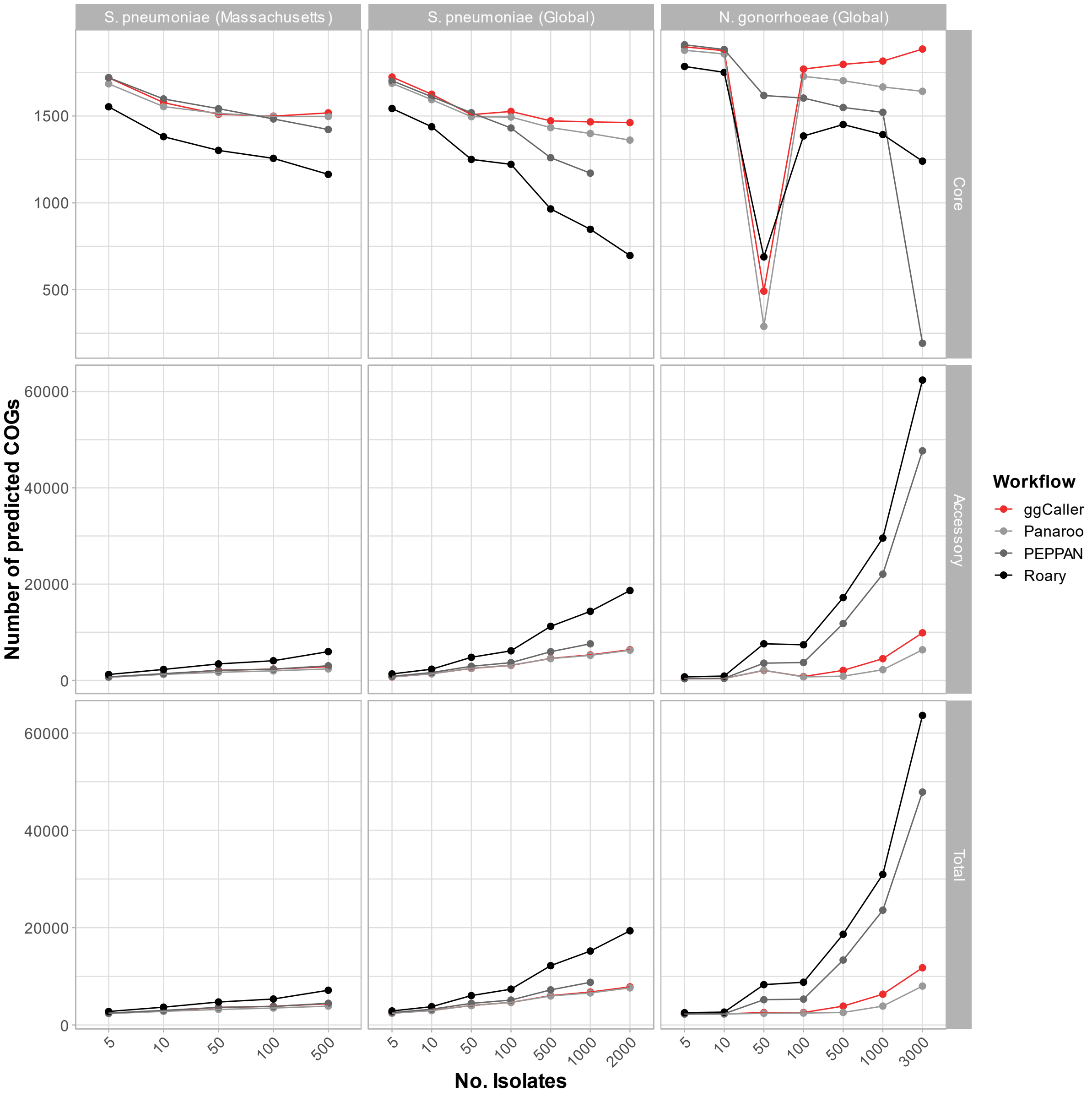

Supplementary Figure 14: Core, accessory and total pangenome size comparison across pangenome analysis tools on three pathogen datasets. Vertical panels describe simulation parameters, vertical panels describe COG frequency; core (99% ≤ x ≤ 100%), accessory (0 ≤ x < 99%) and total (0 ≤ x ≤ 100%). *S. pneumoniae* (Massachusetts), dataset from Croucher *et al*., (2015); *S. pneumoniae* (Global), dataset from Gladstone *et al*., (2019); *N. gonorrhoeae* (Global), dataset from Blackwell *et al*., (2021). The fall in core genome size observed at N=50 genomes for *N. gonorrhoeae* in ggCaller, Panaroo and Roary is likely due to biased sampling of a particular lineage, as it is does not occur at smaller or larger sample sizes.

Supplementary Tables

Supplementary Table 1: Counts of shared and unique False positives (FP) and False negatives (FN) between ggCaller and other gene-prediction tools when applied to pneumococcal capsular biosynthetic operons without fragmentation. FN and FP were calculated based on exact matching to ground truth sequences, meaning 3' and 5' ends must match for identification. If only the 3' end matches between a prediction and ground truth, these sequences are designated as '3' present'. If the prediction does not match the 5' or 3' end of any ground truth, this sequence is designated as '3 absent'.

Comparator	Error type	present	Error presence	Count
GeneMarkS-2 + Panaroo	FP	3' Present	Both	6
			ggCaller	4
			GeneMarkS-2 + Panaroo	3
		3' Absent	Both	12
			ggCaller	13
			GeneMarkS-2 + Panaroo	6
	FN	3' Present	Both	6
			ggCaller	4
			GeneMarkS-2 + Panaroo	3
Prokka + Panaroo	FP	3' Present	Both	5
			ggCaller	5
			Prokka + Panaroo	1
		3' Absent	Both	17
			ggCaller	8
			Prokka + Panaroo	2
	FN	3' Present	Both	5
			ggCaller	5
			Prokka + Panaroo	1

Supplementary Table 2: Core, Accessory and Total pangenome size estimates for simulated bacterial populations. Ground-truth values for each simulation are highlighted in bold. COG categories by frequency; core (99% ≤ x ≤ 100%), Accessory (0 ≤ x < 99%) and total (0 ≤ x ≤ 100%).

Simulation	Workflow	Core COGs	Accessory COGs	Total COGs
G/L=0.1 m=e-14	**Ground-truth**	**2243**	**16**	**2259**
	ggCaller (sensitive)	2147	214	2361
	ggCaller (moderate)	2146	189	2335
	ggCaller (strict)	2145	161	2306
	Prokka + Panaroo (sensitive)	2088	92	2180
	Prokka + Panaroo (moderate)	2088	92	2180
	Prokka + Panaroo (strict)	2074	66	2140
	Prokka + PEPPAN	2066	69	2135
	Prokka + Roary	2013	241	2254
G/L=1 m=e-14	**Ground-truth**	**2143**	**116**	**2259**
	ggCaller (sensitive)	2068	294	2362
	ggCaller (moderate)	2065	253	2318
	ggCaller (strict)	2068	221	2289
	Prokka + Panaroo (sensitive)	1990	196	2186
	Prokka + Panaroo (moderate)	1990	196	2186
	Prokka + Panaroo (strict)	1980	153	2133
	Prokka + PEPPAN	1977	149	2126
	Prokka + Roary	1841	478	2319
G/L=10 m=e-14	**Ground-truth**	**1826**	**433**	**2259**
	ggCaller (sensitive)	1778	565	2343
	ggCaller (moderate)	1772	531	2303
	ggCaller (strict)	1777	488	2265
	Prokka + Panaroo (sensitive)	1700	489	2189
	Prokka + Panaroo (moderate)	1700	474	2174
	Prokka + Panaroo (strict)	1688	447	2135
	Prokka + PEPPAN	1695	433	2128
	Prokka + Roary	1585	686	2271
G/L=10 m=e-15	**Ground-truth**	**1844**	**415**	**2259**
	ggCaller (sensitive)	1802	495	2297
	ggCaller (moderate)	1800	454	2254
	ggCaller (strict)	1801	436	2237
	Prokka + Panaroo (sensitive)	1737	439	2176
	Prokka + Panaroo (moderate)	1735	417	2152
	Prokka + Panaroo (strict)	1723	488	2211
	Prokka + PEPPAN	1708	416	2124
	Prokka + Roary	1668	543	2211
G/L=10 m=e-16	**Ground-truth**	**1771**	**488**	**2259**
	ggCaller (sensitive)	1739	556	2295
	ggCaller (moderate)	1746	507	2253
	ggCaller (strict)	1745	478	2223
	Prokka + Panaroo (sensitive)	1682	496	2178
	Prokka + Panaroo (moderate)	1682	471	2153
	Prokka + Panaroo (strict)	1667	445	2112

	Prokka + PEPPAN	1655	455	2110
	Prokka + Roary	1602	586	2188
Contaminated	**Ground-truth**	**1826**	**433**	**2259**
	ggCaller (sensitive)	1772	763	2535
	ggCaller (moderate)	1768	584	2352
	ggCaller (strict)	1771	540	2311
	Prokka + Panaroo (sensitive)	1715	790	2505
	Prokka + Panaroo (moderate)	1715	501	2216
	Prokka + Panaroo (strict)	1703	453	2156
	Prokka + PEPPAN	1688	780	2468
	Prokka + Roary	1605	1004	2609
Fragmented	**Ground-truth**	**1826**	**433**	**2259**
	ggCaller (sensitive)	1743	1161	2904
	ggCaller (moderate)	1737	822	2559
	ggCaller (strict)	1725	700	2425
	Prokka + Panaroo (sensitive)	1333	1463	2796
	Prokka + Panaroo (moderate)	1333	872	2205
	Prokka + Panaroo (strict)	1322	805	2127
	Prokka + PEPPAN	1531	754	2285
	Prokka + Roary	666	1726	2392

Supplementary Table 3: COG annotation accuracy results for simulated bacterial populations. False positives are COGs that were called by a workflow but were not present in the ground-truth set. False negatives are COGs that were present in the ground-truth set but not called by a workflow. COGs containing errors have an incorrectly predicted gene in at least one individual genome, either a gene that is called when absent, or missed when present.

**Parameters**	**Workflow**	**False positives**	**False negatives**	**≥1 Error**
G/L=0.1 m=e-14	ggCaller (sensitive)	72	160	34
	ggCaller (moderate)	72	162	34
	ggCaller (strict)	72	172	27
	Prokka + Panaroo (sensitive)	84	197	17
	Prokka + Panaroo (moderate)	78	197	17
	Prokka + Panaroo (strict)	71	219	17
	Prokka + PEPPAN	68	253	16
	Prokka + Roary	85	197	78
G/L=1 m=e-14	ggCaller (sensitive)	74	161	22
	ggCaller (moderate)	74	171	21
	ggCaller (strict)	74	180	19
	Prokka + Panaroo (sensitive)	87	194	20
	Prokka + Panaroo (moderate)	82	201	19
	Prokka + Panaroo (strict)	73	229	18
	Prokka + PEPPAN	69	254	8
	Prokka + Roary	93	193	1879
G/L=10 m=e-14	ggCaller (sensitive)	83	170	29
	ggCaller (moderate)	82	186	29
	ggCaller (strict)	82	190	22
	Prokka + Panaroo (sensitive)	81	197	34
	Prokka + Panaroo (moderate)	81	204	33
	Prokka + Panaroo (strict)	68	228	29
	Prokka + PEPPAN	78	259	15
	Prokka + Roary	88	197	1661
G/L=10 m=e-15	ggCaller (sensitive)	78	183	12
	ggCaller (moderate)	78	202	13
	ggCaller (strict)	76	208	14
	Prokka + Panaroo (sensitive)	88	204	23
	Prokka + Panaroo (moderate)	82	217	23
	Prokka + Panaroo (strict)	74	239	21
	Prokka + PEPPAN	72	254	20
	Prokka + Roary	94	203	1716
G/L=10 m=e-16	ggCaller (sensitive)	77	174	15
	ggCaller (moderate)	76	200	14
	ggCaller (strict)	74	216	15
	Prokka + Panaroo (sensitive)	87	201	24
	Prokka + Panaroo (moderate)	81	213	23
	Prokka + Panaroo (strict)	75	233	22
	Prokka + PEPPAN	70	258	12
	Prokka + Roary	89	200	1680
Contaminated	ggCaller (sensitive)	214	174	32

	ggCaller (moderate)	124	190	33
	ggCaller (strict)	119	196	30
	Prokka + Panaroo (sensitive)	321	200	26
	Prokka + Panaroo (moderate)	121	204	25
	Prokka + Panaroo (strict)	96	227	20
	Prokka + PEPPAN	311	257	18
	Prokka + Roary	330	199	108
Fragmented	ggCaller (sensitive)	90	169	425
	ggCaller (moderate)	88	187	421
	ggCaller (strict)	84	197	417
	Prokka + Panaroo (sensitive)	95	191	924
	Prokka + Panaroo (moderate)	84	199	921
	Prokka + Panaroo (strict)	69	228	917
	Prokka + PEPPAN	84	261	509
	Prokka + Roary	158	191	1445

Supplementary Table 4: Comparison of Prokka runtimes and memory usage using default and custom databases. Prokka was run on *Neisseria gonorrhoeae* WHO-M (Genbank Accession: GCA_900087615.2) and *Streptococcus pneumoniae* ATCC 700669 (Genbank Accession: GCA_000026665.1). The *S. pneumoniae* custom database was retrieved from Croucher *et al.* (2015). The *N. gonorrhoeae* custom database was retrieved from Unemo *et al*., (2016).

Species	Annotation database	No. Genomes	No. Gene Annotations (HMM profiles)	Runtime (mins)	Peak Memory (Gb)
Neisseria gonorrhoeae	Default	NA	32148 (2389)	0.57	0.156136
	Custom	14	32056	0.52	0.162148
Streptococcus pneumoniae	Default	NA	32148 (2389)	0.58	0.154436
	Custom	616	1231479	9.47	0.434968

Supplementary Table 5: Simulation parameters using infinitely many genes model. Gene gain and loss rates are measured in per-generation per genome. Gene mutation rate is measured in per-generation per-nucleotide. No. genes is the number of genes simulated (i.e. those in accessory genome), remainder are left as core genes. No. isolates determines how many genomes are simulated. Effective population size determines the population size in terms of the number of gene copies.

Name	Gene gain rate	Gene loss rate	Gene mutation rate	No. Genes	No. Isolates	Effective population size	Additional (custom script used)
G/L=0.1 m=e-14	$1e^{-13}$	$1e^{-12}$	$1e^{-14}$	1000	100	10^6	NA
G/L=1 m=e-14	$1e^{-12}$	$1e^{-12}$	$1e^{-14}$	1000	100	10^6	NA
G/L=10 m=e-14	$1e^{-12}$	$1e^{-13}$	$1e^{-14}$	1000	100	10^6	NA
G/L=10 m=e-15	$1e^{-12}$	$1e^{-13}$	$1e^{-15}$	1000	100	10^6	NA
G/L=10 m=e-16	$1e^{-12}$	$1e^{-13}$	$1e^{-16}$	1000	100	10^6	NA
Contaminated	$1e^{-12}$	$1e^{-13}$	$1e^{-14}$	1000	100	10^6	10kb fragmented Staphylococcus Epidermidis added per genome (insert_random_genome_fragments.py)
Fragmented	$1e^{-12}$	$1e^{-13}$	$1e^{-14}$	1000	100	10^6	Genomes fragmented (fragment_fasta.py)

Supplementary References

Bateman, A., Martin, M. J., Orchard, S., Magrane, M., Agivetova, R., Ahmad, S., Alpi, E., Bowler-Barnett, E. H., Britto, R., Bursteinas, B., Bye-A-Jee, H., Coetzee, R., Cukura, A., da Silva, A., Denny, P., Dogan, T., Ebenezer, T. G., Fan, J., Castro, L. G., … Teodoro, D. (2021). UniProt: the universal protein knowledgebase in 2021. *Nucleic Acids Research*, *49*(D1), D480–D489. https://doi.org/10.1093/NAR/GKAA1100

Baumdicker, F., Hess, W. R., & Pfaffelhuber, P. (2010). The diversity of a distributed genome in bacterial populations. *Https://Doi.Org/10.1214/09-AAP657*, *20*(5), 1567–1606. https://doi.org/10.1214/09-AAP657

Bellman, R. (1958). On a routing problem. *Quarterly of Applied Mathematics*, *16*, 87–90.

Blackwell, G. A., Hunt, M., Malone, K. M., Lima, L., Horesh, G., Alako, B. T. F., Thomson, N. R., & Iqbal, Z. (2021). Exploring bacterial diversity via a curated and searchable snapshot of archived DNA sequences. *PLoS Biology*, *19*(11). https://doi.org/10.1371/JOURNAL.PBIO.3001421

Břinda, K., Baym, M., & Kucherov, G. (2021). Simplitigs as an efficient and scalable representation of de Bruijn graphs. *Genome Biology*, *22*(1). https://doi.org/10.1186/s13059-021-02297-z

Buchfink, B., Xie, C., & Huson, D. H. (2014). Fast and sensitive protein alignment using DIAMOND. *Nature Methods 2014 12:1*, *12*(1), 59–60. https://doi.org/10.1038/nmeth.3176

Cohen, K. A., Abeel, T., Manson McGuire, A., Desjardins, C. A., Munsamy, V., Shea, T. P., Walker, B. J., Bantubani, N., Almeida, D. V., Alvarado, L., Chapman, S. B., Mvelase, N. R., Duffy, E. Y., Fitzgerald, M. G., Govender, P., Gujja, S., Hamilton, S., Howarth, C., Larimer, J. D., … Earl, A. M. (2015). Evolution of Extensively Drug-Resistant Tuberculosis over Four Decades: Whole Genome Sequencing and Dating Analysis of Mycobacterium tuberculosis Isolates from KwaZulu-Natal. *PLoS Medicine*, *12*(9). https://doi.org/10.1371/JOURNAL.PMED.1001880

Collobert, R., Bengio, S., & Mariéthoz, J. (2002). *Torch: a modular machine learning software library*.

Croucher, N. J., Finkelstein, J. A., Pelton, S. I., Parkhill, J., Bentley, S. D., Lipsitch, M., & Hanage, W. P. (2015). Population genomic datasets describing the post-vaccine evolutionary epidemiology of Streptococcus pneumoniae. *Scientific Data 2015*, *2*(1), 1–9. https://doi.org/10.1038/sdata.2015.58

Dagum, L., & Menon, R. (1998). OpenMP: an industry standard API for shared-memory programming. *IEEE Computational Science and Engineering*, *5*(1), 46–55. https://doi.org/10.1109/99.660313

Eddy, S. R. (2009). A new generation of homology search tools based on probabilistic inference. *Genome Informatics. International Conference on Genome Informatics*, *23*(1), 205–211. https://doi.org/10.1142/9781848165632_0019

Ford, L. R., & Fulkerson, D. R. (1962). *Flows in Networks.* Princeton University Press.

Gladstone, R. A., Lo, S. W., Lees, J. A., Croucher, N. J., van Tonder, A. J., Corander, J., Page, A. J., Marttinen, P., Bentley, L. J., Ochoa, T. J., Ho, P. L., du Plessis, M.,

Cornick, J. E., Kwambana-Adams, B., Benisty, R., Nzenze, S. A., Madhi, S. A., Hawkins, P. A., Everett, D. B., … Bentley, S. D. (2019a). International genomic definition of pneumococcal lineages, to contextualise disease, antibiotic resistance and vaccine impact. *EBioMedicine*, *43*, 338–346. https://doi.org/10.1016/j.ebiom.2019.04.021

Gladstone, R. A., Lo, S. W., Lees, J. A., Croucher, N. J., van Tonder, A. J., Corander, J., Page, A. J., Marttinen, P., Bentley, L. J., Ochoa, T. J., Ho, P. L., du Plessis, M., Cornick, J. E., Kwambana-Adams, B., Benisty, R., Nzenze, S. A., Madhi, S. A., Hawkins, P. A., Everett, D. B., … Bentley, S. D. (2019b). International genomic definition of pneumococcal lineages, to contextualise disease, antibiotic resistance and vaccine impact. *EBioMedicine*, *43*, 338–346. https://doi.org/10.1016/j.ebiom.2019.04.021

Gog, S., Beller, T., Moffat, A., & Petri, M. (2014). From theory to practice: Plug and play with succinct data structures. *Lecture Notes in Computer Science (Including Subseries Lecture Notes in Artificial Intelligence and Lecture Notes in Bioinformatics)*, *8504 LNCS*, 326–337. https://doi.org/10.1007/978-3-319-07959-2_28/COVER/

Guennebaud, G., Jacob, B., & others. (2010). *Eigen v3*.

Holley, G., & Melsted, P. (2020). Bifrost: highly parallel construction and indexing of colored and compacted de Bruijn graphs. *Genome Biology*, *21*(1), 249. https://doi.org/10.1186/s13059-020-02135-8

Kallonen, T., Brodrick, H. J., Harris, S. R., Corander, J., Brown, N. M., Martin, V., Peacock, S. J., & Parkhill, J. (2017). Systematic longitudinal survey of invasive Escherichia coli in England demonstrates a stable population structure only transiently disturbed by the emergence of ST131. *Genome Research*, *27*(8), 1437–1449. https://doi.org/10.1101/GR.216606.116

Li, W., & Godzik, A. (2006). Cd-hit: A fast program for clustering and comparing large sets of protein or nucleotide sequences. *Bioinformatics*, *22*(13), 1658–1659. https://doi.org/10.1093/bioinformatics/btl158

Page, A. J., Taylor, B., Delaney, A. J., Soares, J., Seemann, T., Keane, J. A., & Harris, S. R. (2016). SNP-sites: rapid efficient extraction of SNPs from multi-FASTA alignments. *Microbial Genomics*, *2*(4), e000056. https://doi.org/10.1099/MGEN.0.000056/CITE/REFWORKS

Paszke, A., Gross, S., Massa, F., Lerer, A., Bradbury Google, J., Chanan, G., Killeen, T., Lin, Z., Gimelshein, N., Antiga, L., Desmaison, A., Xamla, A. K., Yang, E., Devito, Z., Raison Nabla, M., Tejani, A., Chilamkurthy, S., Ai, Q., Steiner, B., … Chintala, S. (2019). *PyTorch: An Imperative Style, High-Performance Deep Learning Library*. https://doi.org/10.5555/3454287.3455008

Schulz, T., Wittler, R., & Stoye, J. (2022). Sequence-based pangenomic core detection. *IScience*, *25*(6), 104413. https://doi.org/10.1016/J.ISCI.2022.104413

Seemann, T. (2014). Prokka: rapid prokaryotic genome annotation. *Bioinformatics*, *30*(14), 2068–2069. https://doi.org/10.1093/bioinformatics/btu153

Siek, Jeremy., Lee, L.-Quan., & Lumsdaine, Andrew. (2002). *The boost graph library : user guide and reference manual*. Addison-Wesley.

Simonsen, M., Mailund, T., & Pedersen, C. N. S. (2008). Rapid neighbour-joining. *Lecture Notes in Computer Science (Including Subseries Lecture Notes in Artificial Intelligence and Lecture Notes in Bioinformatics)*, *5251 LNBI*, 113–122. https://doi.org/10.1007/978-3-540-87361-7_10/COVER/

Sommer, M. J., & Salzberg, S. L. (2021). Balrog: A universal protein model for prokaryotic gene prediction. *PLOS Computational Biology*, *17*(2), e1008727. https://doi.org/10.1371/journal.pcbi.1008727

Šošić, M., & Šikić, M. (2017). Edlib: a C/C ++ library for fast, exact sequence alignment using edit distance. *Bioinformatics*, *33*(9), 1394–1395. https://doi.org/10.1093/bioinformatics/btw753

Steinegger, M., & Söding, J. (2018). Clustering huge protein sequence sets in linear time. *Nature Communications 2018 9:1*, *9*(1), 1–8. https://doi.org/10.1038/s41467-018-04964-5

Tettelin, H., Masignani, V., Cieslewicz, M. J., Donati, C., Medini, D., Ward, N. L., Angiuoli, S. V, Crabtree, J., Jones, A. L., Durkin, A. S., Deboy, R. T., Davidsen, T. M., Mora, M., Scarselli, M., Margarit Y Ros, I., Peterson, J. D., Hauser, C. R., Sundaram, J. P., Nelson, W. C., … Fraser, C. M. (2005). Genome analysis of multiple pathogenic isolates of Streptococcus agalactiae: Implications for the microbial "pan-genome." *Proceedings of the National Academy of Sciences*, *102*(39), 13950–13955. https://doi.org/10.1073/pnas.0506758102

Tonkin-Hill, G., MacAlasdair, N., Ruis, C., Weimann, A., Horesh, G., Lees, J. A., Gladstone, R. A., Lo, S., Beaudoin, C., Floto, R. A., Frost, S. D. W., Corander, J., Bentley, S. D., & Parkhill, J. (2020). Producing polished prokaryotic pangenomes with the Panaroo pipeline. *Genome Biology*, *21*(1), 1–21. https://doi.org/10.1186/S13059-020-02090-4/FIGURES/7

Unemo, M., Golparian, D., Sánchez-Busó, L., Grad, Y., Jacobsson, S., Ohnishi, M., Lahra, M. M., Limnios, A., Sikora, A. E., Wi, T., & Harris, S. R. (2016). The novel 2016 WHO Neisseria gonorrhoeae reference strains for global quality assurance of laboratory investigations: phenotypic, genetic and reference genome characterization. *The Journal of Antimicrobial Chemotherapy*, *71*(11), 3096–3108. https://doi.org/10.1093/JAC/DKW288
